# Nucleolar localization of the ErbB3 receptor as a new target in glioblastoma

**DOI:** 10.1186/s12860-022-00411-y

**Published:** 2022-03-07

**Authors:** Marzia Tagliaferro, Paolo Rosa, Gian Carlo Bellenchi, Daniela Bastianelli, Rosa Trotta, Claudia Tito, Francesco Fazi, Antonella Calogero, Donatella Ponti

**Affiliations:** 1 Department of Medical-Surgical Sciences and Biotechnologies, University of Rome La Sapienza, Corso della Repubblica 79, 04100 Latina, Italy; 2 Institute of Genetics and Biophysics “Adriano Buzzati Traverso” CNR, 80131 Naples, Italy; 3 Fondazione Santa Lucia IRCCS, 00143 Rome, Italy; 4 Department of Systems Medicine, University of Tor Vergata, 00133 Rome, Italy; 5 Istituto Chirurgico Ortopedico Traumatologico, 04100 Latina, Italy; 6 Laboratory of Tumor Inflammation and Angiogenesis, Center for Cancer Biology (CCB), VIB, Leuven, Belgium; 7 Laboratory of Tumor Inflammation and Angiogenesis, and Department of Oncology, KU Leuven, Leuven, Belgium; 8 Department of Anatomical, Histological, Forensic and Orthopaedic Sciences, Sapienza University of Rome, 00185 Rome, Italy

**Keywords:** ErbB3, Neuregulin, Nucleolus, UBF, Nucleolin, Glioblastoma, Actinomycin D

## Abstract

**Background:**

The nucleolus is a subnuclear, non-membrane bound domain that is the hub of ribosome biogenesis and a critical regulator of cell homeostasis. Rapid growth and division of cells in tumors are correlated with intensive nucleolar metabolism as a response to oncogenic factors overexpression. Several members of the Epidermal Growth Factor Receptor (EGFR) family, have been identified in the nucleus and nucleolus of many cancer cells, but their function in these compartments remains unexplored.

**Results:**

We focused our research on the nucleolar function that a specific member of EGFR family, the ErbB3 receptor, plays in glioblastoma, a tumor without effective therapies. Here, Neuregulin 1 mediated proliferative stimuli, promotes ErbB3 relocalization from the nucleolus to the cytoplasm and increases pre-rRNA synthesis. Instead ErbB3 silencing or nucleolar stress reduce cell proliferation and affect cell cycle progression.

**Conclusions:**

These data point to the existence of an ErbB3-mediated non canonical pathway that glioblastoma cells use to control ribosomes synthesis and cell proliferation. These results highlight the potential role for the nucleolar ErbB3 receptor, as a new target in glioblastoma.

**Supplementary Information:**

The online version contains supplementary material available at 10.1186/s12860-022-00411-y.

## Background

In the eukaryotic cells the production of ribosomes takes place in the nucleolus, a specialized subnuclear compartment where the RNA Polymerase I (RNA Pol I) catalyses the ribosomal RNA synthesis (rRNA). The activity of this enzyme is under the control of two main factors, UBF and SL1 both components of the complex that stabilizes RNA Pol I on rDNA promoter [1, 2]. Ribosomal RNA is synthesised as precursor 47S pre-rRNA which is subjected to specific and highly regulated processing steps that allow to mature 18S, 5.6S and 28S RNAs. During ribosomal RNA processing, ribosomal proteins are incorporated into the pre-ribosomal subunits to form the mature 40S and 60S subunits. Tumor suppressors, oncogenes and alternatively deregulated upstream signalling pathways can directly influence the RNA polymerase I activity inducing hyper activation of rRNA transcription in cancer cells. Thus, the control of ribosome assembly and protein synthesis is essential for the survival of cancer cells. Tumor suppressors such as retinoblastoma (pRB) and p53 negatively regulate RNA polymerase I and interfere with the assembly of transcriptional machinery on the rDNA promoter. In this context, during any sort of stress condition the alternative reading frame protein p14ARF subtracts MDM2, an E3 ubiquitin ligase, from the interaction with p53 in manner to free and stabilise p53 level in the nucleolus of the cell where it inhibits RNA polymerase I activity [3]. Recently we demonstrated the involvement of EGR1, an early gene normally induced after stress signal, as negative regulator of RNA polymerase I [4]. Nucleolar function of EGR1 is strictly linked to the expression of nucleolar proteins such as nucleophosmin (B23) and alternative reading frame (p14ARF). These findings demonstrated the action of a new specific nucleolar network EGR1-B23-ARF, alternative to p53, that negatively regulates RNA polymerase I in cancer cells [5]. Nucleolar activity is also influenced by the interaction between pathways activated from extracellular signals in order to coordinate ribosome synthesis and cell proliferation [6]. Indeed, proteomic analysis has shown that a variety of proteins involved in ribosome biogenesis and proteins that have extra nucleolar function with effects on cell cycle and apoptosis localize in the nucleolus [7–11] are chromatin modulators and transcriptional regulators that influence nucleolar activity [12]. The EGFR/ERBB/HER proto-oncogene family consists of four type-I tyrosine kinase receptors, ErbB1–4 and many studies indicate, the presence in the nucleus and nucleolus of several ErbB receptors [13–15]. Generally, these receptors explain their function on the cell membrane where, after the binding with growth factors or neuregulin undergo to homo or hetero oligomerization with the activation of the intrinsic tyrosine kinase activity and the subsequent recruitment of proteins involved in the cytoplasmic signalling pathways. This signalling generates cross-talks with multiple cellular processes such as proliferation, cell mobility and apoptosis [14]. Since the EGFR signalling promotes proliferation in tumor the EGFR receptors often represent a marker of resistance with poor prognosis. All EGFR family members have been described in the nuclei of various normal and/or tumoral cell lines, either as full length receptors either as alternatively generated nuclear variants or as proteolytically cleaved proteins. While ErbB1 and ErbB2 were identified in the nucleus as full-length molecules/proteins [15–17], ErbB4 and ErbB3 were found in the nucleus as truncated isoforms. In particular a shorter 80 kDa ErbB3 variant regulates the expression of cyclin D1 and relocalizes in the nucleolus of H358 cells after p14ARF over expression [13, 18]. Our hypothesis is that several of these EGFR receptors, could directly influence the nuclear and/or nucleolar activity in cancer cells. Particularly we focused our attention on the nucleolar function of ErbB3 receptor in glioblastoma. ErbB3 is a 180-kDa transmembrane glycoprotein composed of three regions, a NH2-terminal extracellular ligand-binding region, a transmembrane domain, and an intracellular region containing the COOH-terminal [19]. It is expressed in normal brain preferentially as full length although recently it has been identified also as truncated isoforms of 55 kDa and 21 kDa which presumably include only the C-terminal domain [20].

The first hypothesis was that ErbB3 could be subjected to stepwise proteolysis. However the absence of metalloproteinase proteins’ cleavage sites has suggested that the nuclear protein could be the result of an alternative splicing [21].

Primary ligands of ErbB3 are the members of the neuregulin (NRG) family. After NRG activation, the ErbB3 receptor physically associate with other ErbB family members, to form heterodimers or higher-order oligomers [22].

The presence of ErbB3 in the nucleolus has been described in several cancer cell lines but its function in this compartment remains unexplored at moment. We further explored the ErbB3 nucleolar function in glioblastoma describing, for the first time, the presence of the 50 kDa variant of ErbB3 receptor in the nucleolus of glioblastoma cells. Under neuregulin stimulation it relocalizes to the cytoplasm with a concomitant increasing of the 47S pre-rRNA synthesis. The impairment of ErbB3 trafficking by silencing or promoting nucleolar stress, determines a cell cycle block and affects cell survival. This nuclear/cytoplasm traffic of ErbB3 observed in vitro was also confirmed *in* ex-vivo in glioblastoma primary cells*.* Our findings support the hypothesis for the existence, of an ErbB3 alternative pathway controlling cell proliferation of glioblastoma cells that opens new perspectives to investigate therapeutic approaches aimed to modulate ErbB3 in glioblastoma.

## Results

### Nuclear-cytoplasmic localization of ErbB3 receptor

**Fig. 1:**
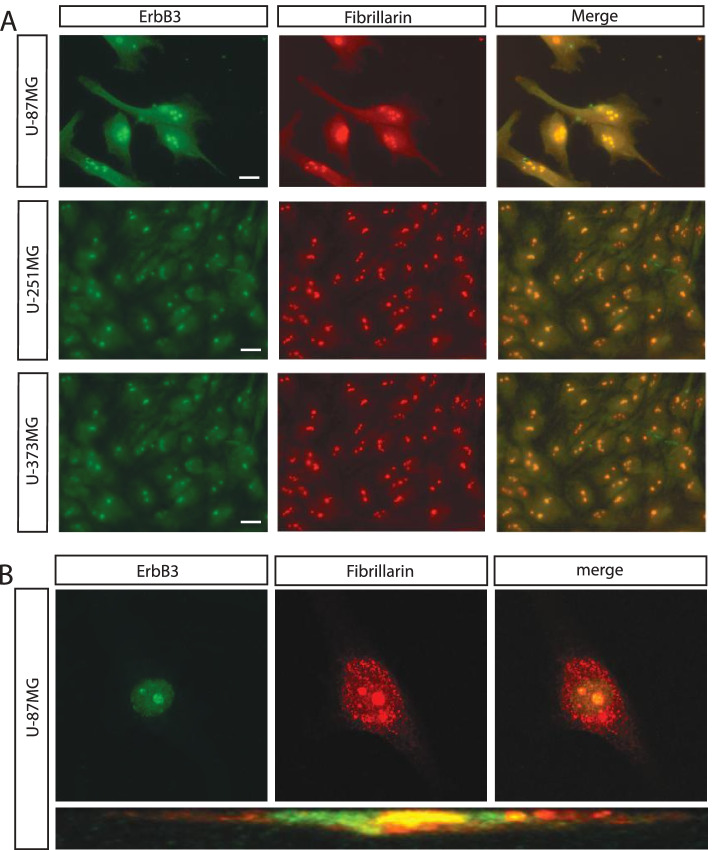
ErbB3 localizes in the nucleolus of ovarian and glioblastoma cell lines. Immunofluorescence analysis for ErbB3 (green) and Fibrillarin (red) after 24 h of serum starvation (0.2% FBS), in the glioblastoma cell lines U-87MG, U-251MG and U-373MG (**A**). Confocal magnifications and relative lateral sections are shown for U-87MG cells (**B**). ErbB3 staining was performed by using rabbit polyclonal anti-ErbB3 sc-285. The colocalization is shown in yellow. Scalebar represent 10 μm

Glioblastoma is a common adult brain tumor in which the enhanced malignant potential correlates with the expression of ErbB1 [14, 15]. Although there is an extensive literature concerning the role of ErbB1 in glioblastoma, few studies have investigated the contribution to glioblastoma development by the other members of the ErbB family, such as ErbB3. In tumors ErbB3 localizes in the nucleus and is often over expressed when cells escape from the antiproliferative effects of ErbB1 inhibitors, thus suggesting its possible involvement in resistance development. Little is known regarding the role of ErbB3 in the nucleus. To address this point we investigate ErbB3 localization by immunostaining with anti ErbB3 (named SC-285) antibody, that recognise specifically the C-terminal region of the receptor. Immunostainings were performed in different glioblastoma cell lines such as the U-87MG, known to express ErbB3, the U-251MG and the U-373MG (Fig. 1A). We also observed a strong signal in HeLa, a uterine cervix cancer cell line characterised by the absence of ErbB3 receptor on the cell surface [23] and in MCF-7 known to express ErbB3 on the plasma membrane [24] (Fig. S1). We found that ErbB3 colocalizes with the nucleolar markers fibrillarin or the RNA polymerase I subunit RPA40 in all the glioblastoma cell lines analysed although with different intensity of the signal while in MCF-7 ErbB3 appears expressed on the cell surface and in both the nucleus and in the nucleoli of the cells. The ErbB3 nucleolar localization was further confirmed by confocal imaging using conventional and lateral resolution for U-87MG and HeLa cells (Fig. 1B and Fig. S1).

As expected any nucleolar staining was detected when the immunofluorescence was performed in absence of primary antibody and without permeabilization (Fig. S2A,B). U-87MG cells were also stained for ErbB3 by using a different source of antibody (named RTJ2). The nucleolar staining and the colocalization with fibrillarin was confirmed also in this case (Fig. S3).

**Fig. 2:**
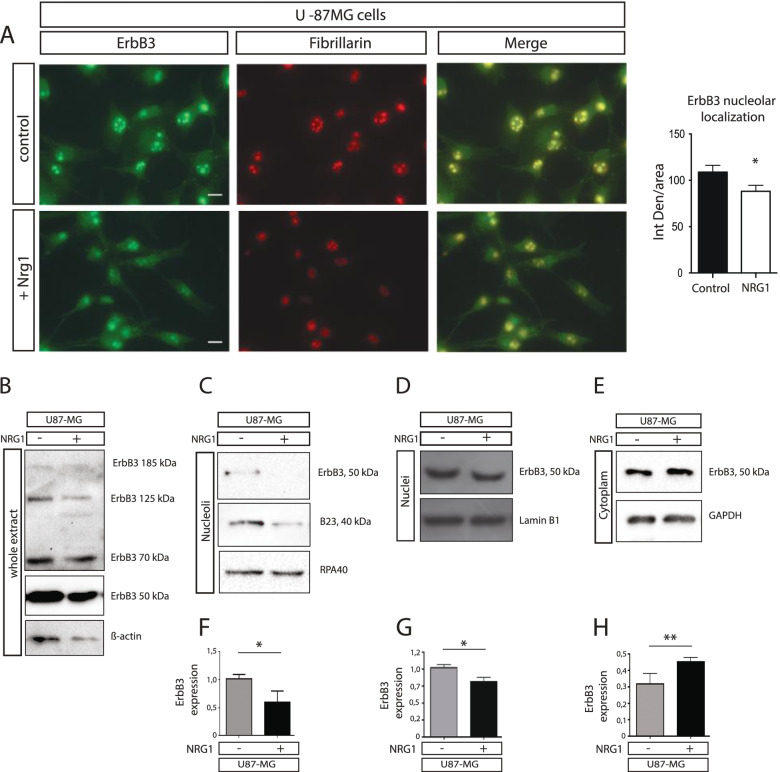
Nucleolar localization of ErbB3 is under the control of Neuregulin1. Immunofluorescence analysis for the ErbB3 receptor (green) and B23 (red) in U-87MG cells before and after Neuregulin1 treatment (**A**). The colocalization is shown in yellow. Scalebar represent 10 μm. Densitometric quantification of the ErbB3 signal in the nucleoli was performed by Image J. U-87MG cell lines were subjected to cell fractionation after 15 min of Neuregulin1 stimulation and analysed by western blot. Whole extract (**B**), purified nucleoli (**C**), Nucleoplasm (**D**) and cytoplasmic extracts (**E**) were probes with anti ErbB3, anti B23 (only for nucleoli) and specific antibodies for each compartments corresponding to anti Pol I subunit RPA40 for the nucleoli; anti GAPDH for the cytoplasm and anti lamin B1 for the nuclei. The proteins amount loaded in each lane were: whole extract 80 μg, nucleoli, 4 μg; nuclei 10 μg; cytoplasm 40 μg. The quantification data shown in F-H are the average of three independent experiments and significant results are highlighted with asterisks (* *p* < 0.05, ** *p* < 0.01)

Since NRG1 has a crucial role in ErbB3 activation [25] we investigate if and how it may control ErbB3 nucleolar localization. To this purpose U-87MG cells were stimulated with NRG1 (15′) and then analysed for ErbB3 expression. We found that NRG1 treatment induces a significant reduction of nucleolar ErbB3 in U-87MG (Fig. 2A). The nucleolar marker nucleophosmin (B23) was also reduced. To investigate if the reduction in the immunofluorescence signal after proliferative stimuli was effectively associated to a reduced expression of nucleolar ErbB3, we analysed the expression of the protein in whole extract, nucleoplasm, cytoplasm and nucleoli after cell fractionation in U87-MG cell lines (Fig. 2B-E). In all samples the prevalent form of ErbB3 corresponded to the short 50 kDa variant, although in whole lysate weaker signals, for the ErbB3 fragments at 125 kDa and 70 kDa, were visible. The full length ErbB3 receptor of 185 kDa appeared instead always as a faint band (Fig. 2B). The short 50 kDa ErbB3 variant was also the predominant form in MCF-7 cells (S4). The nucleolar expression of the 50 kDa ErbB3 receptor was strongly reduced after NRG1 treatment (Fig. 2C,F), while its levels in the nucleus (Fig. 2C,G) and in the cytoplasm (Fig. 2D,H) were slightly but significantly changed, after NRG1 treatment. A similar trend was observed in HeLa cells were we found up to a 20% average reduction of ErbB3 after NRG1 stimulation in the nucleolus of this cells (Fig. S5A-F), thus confirming the immunofluorescence data. These findings suggest that NRG1 drives the shuttling of the 50 kDa ErbB3 variant from the nucleolus to the cytoplasm both in U87-MG and HeLa cells.

### ErbB3 interacts with nucleolar proteins

**Fig. 3:**
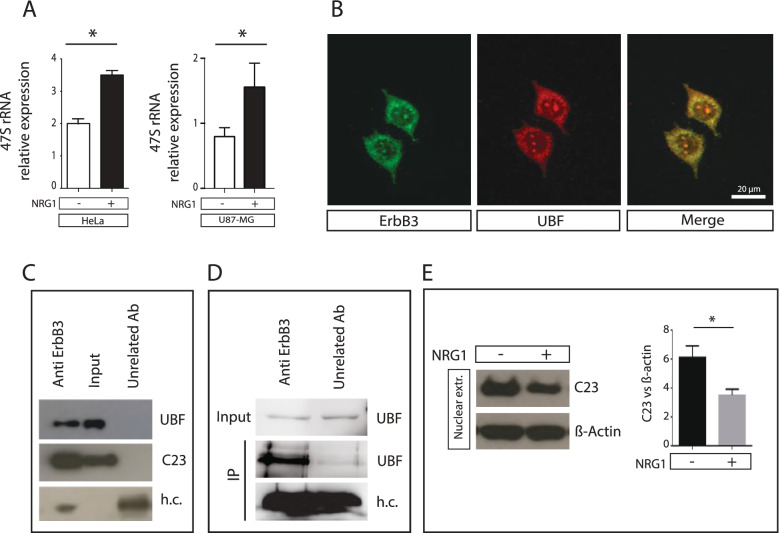
ErbB3 interacts with the nucleolar proteins UBF and C23. The expression of 47S pre-rRNA precursor in HeLa and U-87MG cells was analyzed by qPCR before and after NRG1 treatment (**A**). HeLa cells were immunostained with anti ErbB3 (green) and anti UBF (red). The colocalization is shown in yellow (**B**). Scalebar represent 5 μm. Nuclear extracts from HeLa and U-87MG cells were immunoprecipitated with anti ErbB3 and analyzed by western blot with anti UBF (**C**, **D**) and anti C23 antibodies (**C**). The heavy chains are shown as well. The nuclear localization of C23 was investigate by western blot in nuclear extracts before and after neuregulin1 treatment (**E**). The densitometric quantification of the C23 signal was performed by Image J (**E**, right) . Data are the average of three independent experiments and the significant results are highlighted with asterisks (* *p* ≤ 0.05)

The nucleolus is a subnuclear compartment devoted to ribosomes biogenesis. Ribosome synthesis requires 80% of cell energy. For this reason this process is tightly regulated via extracellular and intracellular signalling that in the nucleolus control the synthesis of the 47S pre-rRNA through the modulation of the RNA polymerase I activity [1, 2]. Since all the proteins that localize in this structure are somehow linked to ribosome biogenesis, we investigated the involvement of ErbB3 in this process by analysing through qPCR the levels of 47S pre-rRNA. Interestingly the reduction of nucleolar ErbB3 expression correlates with an increased expression of the 47S ribosomal precursor in both HeLa and U-87MG cell lines (Fig. 3A). Thus we hypothesised that ErbB3 could regulate RNA Polymerase I by modulating the Upstream Binding Factor (UBF) activity, a key component controlling the transcription of 47S pre-rRNA. To address this hypothesis we searched for a possible interaction between ErbB3 and UBF through experiments of immunocolocalization and immunoprecipitaton. To this purpose we used HeLa cells since they have higher number of nucleoli if compared to other cell lines and represent a good model to dissect nucleolar interactions. Here ErbB3 colocalizes in the nucleolus with UBF (Fig. 3B) in absence of NRG1 stimulation. When we immunoprecipitated the ErbB3 complex with an anti ErbB3 antibody we found an enrichment also in UBF, while no enrichment was detected when we used an unrelated antibody either in HeLa either in U-87MG (Fig. 3 C,D). Since other members of EGFR family (ErbB1 and ErbB2) interact with the nucleolar protein nucleolin (also known as C23) [26, 27], we investigate and confirmed that C23 is also part of the ErbB3-UBF complex (Fig. 3C). These data suggest that ErbB3 is interested in multiple interactions in the nucleolus. Since C23 is a nucleolar protein involved in ribosomal precursor processing and shuttles from the nucleolus to the cell membrane [26, 27] under proliferation stimuli, we verify if it undergoes shuttling upon NRG1 stimulation. To this purpose we profiled its expression in nuclear extracts. After NRG1 treatment the C23 protein level decreases in the nucleus (Fig. 3E) as described for ErbB3.

### ErbB3 and nucleolar stress

Nucleolar metabolism is strictly linked to the rate of cell proliferation, besides cancer cells have a higher number of the nucleoli when compared to non-pathological cells [3]. In additionthe size of the nucleoli is proportional to the rate of ribosomes biosynthesis and any stressful condition such as that obtained by a chemotherapy agent causes reduction of ribosome biogenesis and decreases their size.

**Fig. 4:**
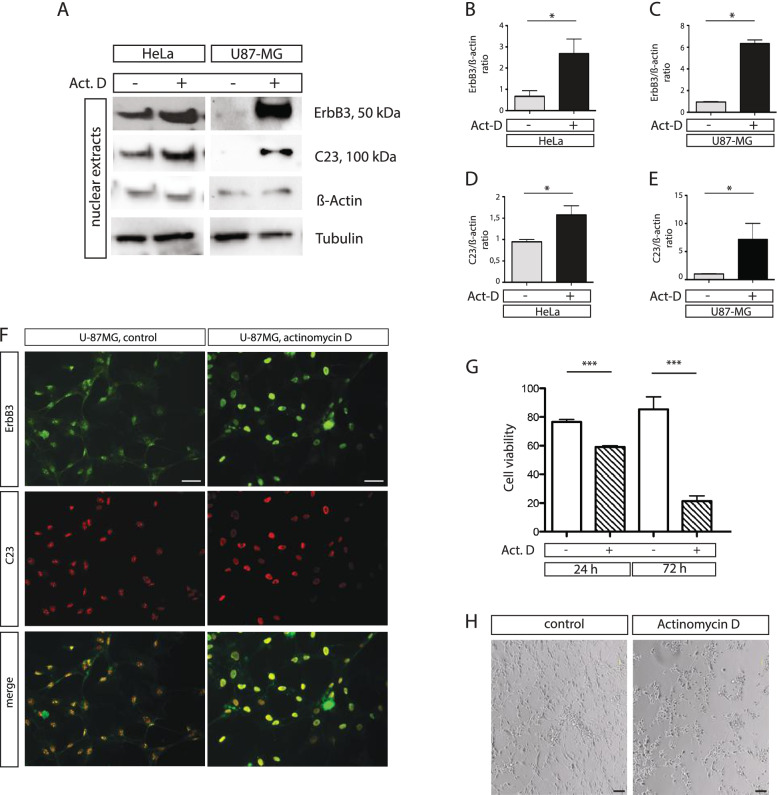
Nucleolar stress induces nuclear ErbB3 accumulation and affects cell viability. Nuclear extracts obtained from HeLa and U-87MG cells treated with 0,04 μg/ml of actinomycin D were analysed by western blot for the expression of ErbB3 and the nucleolar marker C23 (**A**). Densitometric quantification of the ErbB3 and C23 signals was performed with the Image J software (**B-E**). ErbB3 re-localizes to the nucleoplasm upon low Actinomycin treatment in U-87MG cell (**F**). Scalebar represent 50 μm. U-87MG cell survival, measured by trypan blue, was strongly affected after actinomycin D treatment (**G**). Remarkable changes are evident, after 72 h exposure to actinomycin D and overall cell shape and density (**H**). Scalebar represent 100 μm. Data are the average of three independent experiments and significant results are highlighted with asterisks (* *p* < 0.05, *** *p* < 0.001)

To investigate the involvement of 50 kDa ErbB3 in the nucleolar metabolism we used Actinomycin D, a molecule that at low dose induces nucleolar stress [28]. HeLa and U-87MG cell lines were both treated with Actinomycin D for 1 h and the nuclear extracts, once purified, were analysed by western blot. A significant increasing of the short variant ErbB3 was detected in the nucleoplasm of both cell lines after treatment (Fig. 4A-C). Under nucleolar stress also C23 accumulates in the nucleus, suggesting a possible functional link between these proteins (Fig. 4A-F). To investigate if the interference on ErbB3 nuclear-cytoplasmic traffic has effect on cell viability we measured cell viability by trypan blue assay in U-87MG cells after Actinomycin D low dose treatment for 24 h and 72 h. We observed a clear effect on cell viability with increased cell death from 1.4 to 4 folds respectively at 24 h and 72 h (Fig. 4G-H). Furthermore, Actinomycin D induced changes in the growth medium pH as a result of a lower metabolic activity induced by nucleolar stress (Fig. S6).

### ErbB3 silencing affects survival of glioblastoma cell line

**Fig. 5:**
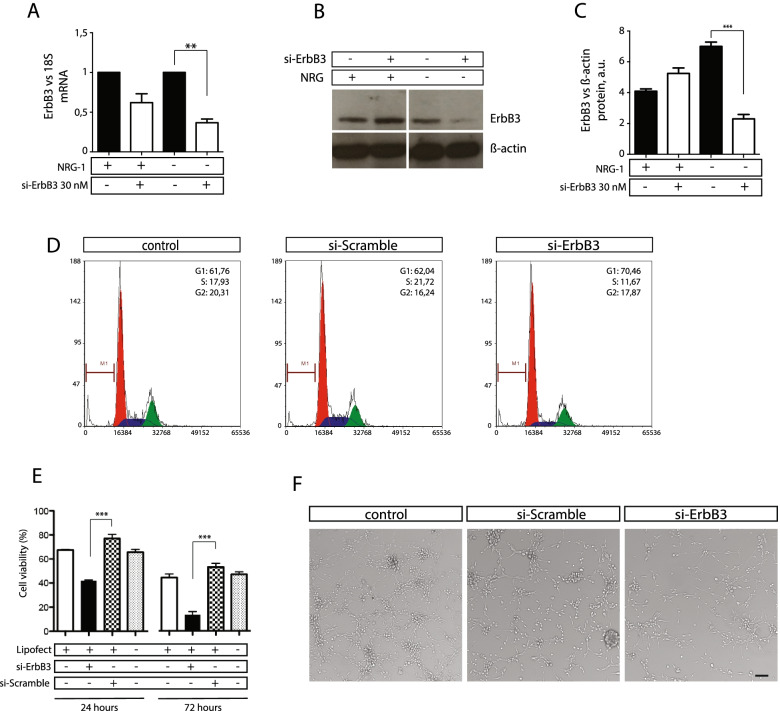
ErbB3 silencing affects cell cycle progression and cell survival. U-87MG (**A-C**) cells were transfected with 30 nM of siErbB3 or siScramble as control. The efficiency of ErbB3 silencing was analysed at mRNA level, by qPCR (A) and at protein level by western blot (**B**). The densitometric quantification of ErbB3 signal, in western blots, was performed by using the Image J software (**C**). U-87MG cells, transfected with siErbB3 at 30 nM for 24 h, show a decrease percentage of cells in S phase after cytofluorimetry analysis (**D**). U-87MG cell viability was analysed by trypan blue after 24 and 72 h of treatment with 30 nM of siErbb3 (**E**). Representative images of untreated cells and cells treated with 30 nM of siErbB3 or siScramble are shown (**F**). Scalebar represent 100 μm. Data are the mean of three independent experiments. Significant results are highlighted with asterisks (** *p* < 0.01, *** *p* < 0.001)

To better understand the role of ErbB3 in the nucleolus we knocked down ErbB3 by silencing its expression through specific siErbB3 that targets the exon 2 of the *ErbB3* gene. To this purpose 30 nM of siErbB3 was used to obtain a significant reduction of both mRNA and protein in U-87MG cells (Fig. 5A-C). ErbB3 silencing elicited an average reduction of 30–60% protein levels, if compared with a scramble siRNAs (Fig. 5C). In addition ErbB3 silencing resulted in a significant reduction of cell cycle at S phase (11%) when compared to si-Scramble (21%) (Fig. 5D) and in the reduction of cell viability of 1,6 folds and 3,3 folds at 24 h and 72 h respectively if compared to control samples (Fig. 5E,F). This data supports our hypothesis that the ErbB3 variant could be involved in a proliferative pathway crucial for glioblastoma cells. Silencing of ErbB3 affects cell viability thus underlining the role of nucleolar ErbB3 as a potential target in glioblastoma.

#### Nucleolar localization of ErbB3 in a primary culture of glioblastoma affected patients

**Fig. 6:**
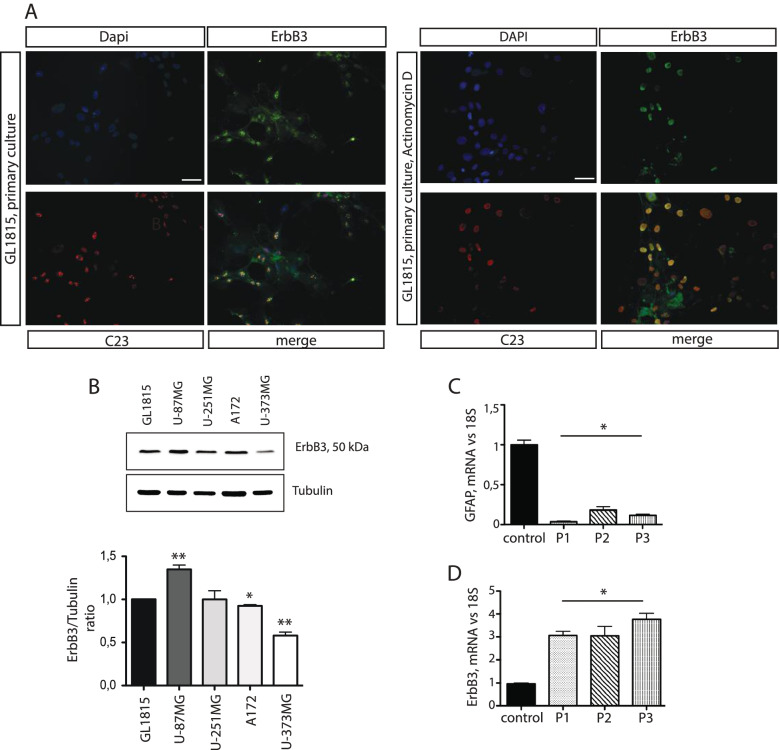
Nucleolar localization of ErbB3 in primary cells of glioblastoma. ErbB3 localizes in the nucleolus of cells derived from primary glioblastoma culture (left panel) and re-localizes to the nucleoplasm upon low Actinomycin treatment (right panel) (**A**). Scalebar represent 50 μm. The specificity of the anti ErbB3 antibody was verified in a primary culture of glioblastoma (GL18–15), in U-87MG, U-251MG, A172 and U-373MG (**B**). The quantification of the signal is shown in the histogram (B, lower panel). The proteins amount loaded in each lane was 80 μg. The expression of GAFP (**C**) and ErbB3 (**D**) were analyzed by qPCR from mRNA purified from patient biopsies. Data are the average of three independent experiments. Significant results are highlighted with asterisks (* *p* < 0.05)

The nucleolar localization of ErbB3 was also investigated in glioblastoma primary cells obtained from biopsies of affected patients. Here ErbB3 localizes in the nucleolus as already shown for U-87MG. Treatment with low doses of actinomycin D re-localizes ErbB3 in the nucleoplasm together with C23 (Fig. 6A). The expression of the 50 kDa ErbB3 was also verified by western blot in cellular extracts obtained from glioblastoma primary cell cultures and from U-251MG, A-172 and U-373MG cell lines. All cell lines express the same variant of ErbB3 (Fig. 6B) although at different levels, as shown by the quantification (histogram in Fig. 6B lower panel). Patients biopsies immunostained for ErbB3 show a heterogeneous localization spanning from nucleolus to cytoplasm (Fig. S7). These cells show also the typical reduction of GFAP expression, a hallmark of cancer cells (Fig. 6C) associated to the overall increase in ErbB3 synthesis, as shown from qPCR analysis performed on total RNA extracted from patient biopsies (Fig. 6D).

## Discussion

In several tumors, survival and proliferation are driven by autocrine and paracrine activity of cancer cells. Several reports have identified neuregulin ligands as potential endogenous factor that allows the activation of specific components of EGFR family such as ErbB3 [29–31] and over expression of ErbB3 has been frequently detected in a variety of cancers, including those of the breast, colon, stomach, ovary, pancreas and brain [19, 32–37]. Glioblastoma multiforme is the IV grade glioma that represents the most prevalent malignancy tumor in the brain. It is characterized by aberrant activation of signaling pathways mediated by receptor tyrosine kinases [38, 39]. Here, ErbB3 overexpression predicts poor prognosis in affected patient and is often associated with resistance to EGFR-target therapy [40]. Several evidence, have shown the involvement of ErbB3 receptors in extra membrane functions in both cancer and normal tissues [20]. In the nucleus a short variant of ErbB3 controls the expression of Cyclin-D1 [13], thus strengthening the hypothesis that in the nucleolus it could have the role of neutralize its pro-proliferative effect when required by the cell. In addition, the nucleolus has been shown to represent a special nuclear hub aimed to mask the function of crucial proteins when needed. Following this data, we can easily hypothesize that ErbB3 could shuttle between the nucleolus and cytoplasm following specific “environmental” cues. However, this hypothesis, does not exclude a possible active function of ErbB3 in the nucleolar compartment. Indeed, it could interact with nucleolar components and modulates nucleolar metabolism and ribosome biogenesis. In the present study, we identified for the first time that an already described shorter variant of ErbB3 [21] undergoes relocalization in glioblastoma cancer cells. Here ErbB3 traffic could have two functions: i) to activate the RNA Polymerase I; ii) to confer a gain function to the ErbB3 receptor after the transit through the nucleolus.

We proposed a non-canonical activation pathway for the shorter variant, that does not required the NRG1 binding and ErbB3 heterodimerization. An intriguingly hypothesis is that higher level of NRG1 could activate an alternatively splicing of the full-length receptor. Indeed the silico analysis of the ErbB3 locus predicts 15 different mRNAs, 14 alternatively spliced variants and one unspliced form [21]. Once produced this shorter variant undergoes then nuclear localization driven by an alternative NLS located between the amino acids 1118–1127 at his C-terminal region [41, 42].

**Fig. 7:**
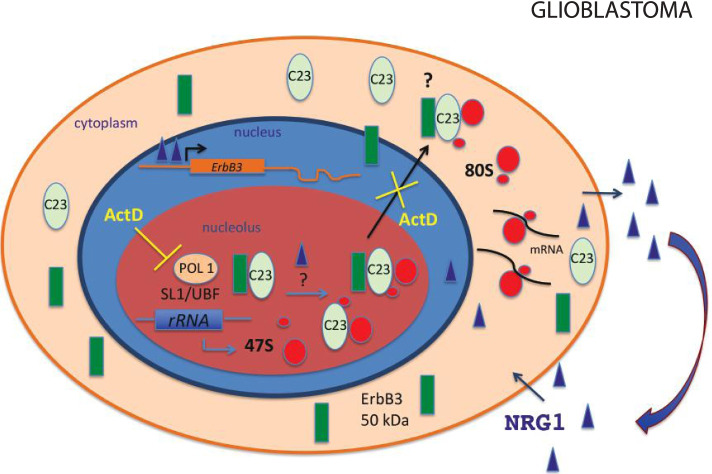
Scheme of non-canonical ErbB3 trafficking between cell microdomains

Our finding shows a significant reduction of cells in S phase after ErbB3 silencing, suggesting this approach as a new potential therapeutic treatment alone or in combination with other therapies, to counteract tumor progression in glioblastoma. The most obvious advantage for the ErbB3 translocation into the nucleus is that it can deliver specific signals avoiding the interference of common pathways shared by other membrane receptors. It is known that ErbB3 binds the DNA and interacts with transcription factors involved in cell survival and proliferation [42]. We hypothesized that ErbB3 transit in the nucleolus represents an alternative strategy to influence the RNA polymerase I activity. A possible scenario could be that in conditions of serum starvation the tandem ErbB3/C23 interferes with RNA Polymerase I activity and 47S rRNA synthesis with the reduction of the cell proliferation. When the concentration of NRG1 increases, (overexpressed in high grade glioblastoma), the ErbB3/C23 could be released from the complex and moves to the cytoplasm, with positive effect on cell proliferation. The binding of actinomycin D interferes with this mechanism and induce nuclear accumulation of ErbB3/C23 with a reduction of ribosome biogenesis and block of cell proliferation (Fig. 7). Nucleolin has been shown to be overexpressed in glioblastoma where its cytoplasmic localization increases proportionally to the tumor grade [43], thus the interaction between ErbB3 and C23 could have a functional related to tumor progression. This scenario might explain the nucleolar localization of ErbB3 in primary cells of glioblastoma and the partial nucleolar localization observed in patient biopsies. This last data supports our idea that nucleolar localization of ErbB3 may be important also in vivo, although other factors may control or be involved in this process. Regarding a possible therapeutic approach based on a low dose Actinomycin D treatment it is worth mentioning a recent work where the treatment with Actinomycin D at 0.05 mg/Kg counteracts recurrent glioblastoma [44]. Our work goes in the same direction and our findings support nucleolar ErbB3 as a new player in glioblastoma cancer cells. Thus, drugs involved in nucleolar stress such as Actinomycin D, could be introduced in combination with ErbB3 target therapies as new strategy for targeting these tumors.

## Conclusions

The modulation of the nucleolar ErbB3 could pave the way towards potential anti-cancer backup strategies and could represent a possible novel pharmacological approach to counteract cell proliferation in tumours.

## Materials

### Cell culture

The MCF-7, HeLa, U-87MG, U-251MG, A172 and U-373MG cell lines, were provided by American Type Collection, Rockville, MD (ATCC). The cell lines were cultivated in DMEM medium or in DMEM-F-12. The mediums in both cases were supplemented with 10% heat inactivated FBS (Sigma-Aldrich St. Louis, Mo, USA), 2 mM glutamine (GIBCO), 1 mM sodium pyruvate (SIGMA), MEM non-essential amino acids (INVITROGEN), and antibiotics (100 IU/ml penicillin, 100 IU/ml streptomycin) (GIBCO). Cells were maintained in humidified incubators with 5% CO_2_ at 37 °C. GL18–15 primary culture was derived from a patient who was diagnosed with glioblastoma (WHO grade IV) and underwent surgical resection. The intraoperatory sample was placed in DMEM/F12 medium supplemented with 10% FBS at 4 °C. Then, GBM tissues were minced with scalpel blades and mechanically dissociated with sterile glass pipettes. Before plating, the cell suspension was filtered twice with a 100 μm and again with a 70 μm cell strainer and centrifuged at 200 x g for 5 min at room temperature. Medium was half changed every 4 days and the cells were subcultured only when they reached total confluency. The cells were starved in 0.2% FBS cultivation medium overnight and stimulated with 4 ng/ml NRG1 for 15 min at 37 °C. *Nucleolar stress*. The cells at 80% of confluence were incubated with 0,04 μg/ml of Actinomycin D (A9415, Sigma) for 1 h at 37°. For experiments of viability the treatment was performed for 24 and 72 h at 37 °C. Cell viability was determined by loading 10 μl of the cell suspension into countess cell counting chamber slides that were read by the countess automated cell counter (INVITROGEN, Carlsbad, CA). The cells were diluted 1:1 with trypan blue. Images were acquired with Nikon Eclipse Ni motorized microscope. Three independent experiments were performed in triplicate.

### Immunofluorescence analysis

The immunofluorescence analysis was performed according to Ponti et al. [4, 5]. Briefly: the cells were cultured in 10% FBS or 0.2% FBS for 18 h than washed with PBS, fixed for 15 min with 4% paraformaldehyde (SIGMA-ALDRICH-Aldrich St. Louis, Mo, USA). The cells were treated with 0.5% Triton X-100 for 10 min and blocked for 40 min with 0.2% gelatine. All incubations with primary antibodies were performed in 0.5% PBS-Triton X-100 overnight at 4 °C. The following primary antibodies were used for immunofluorescence: rabbit polyclonal against anti-ErbB3 (sc-285, SANTA CRUZ BIOTECHNOLOGY, Dallas, Tx, USA), mouse monoclonal antibody anti-fibrillarin (ab4566, Abcam, Cambridge, MA, USA), rabbit polyclonal antibody anti-fibrillarin (PA5–29801, Thermo Fisher Scientific Waltham, Massachusetts, USA) mouse monoclonal antibody anti-B23 (ab10530, Abcam, Cambridge, MA, USA) and mouse monoclonal anti-UBF antibody (sc-13,125, SANTA CRUZ BIOTECHNOLOGY, Dallas, TX, USA). Primary antibodies were diluted at 1:200. Cells were incubated with secondary antibodies, Alexa-Fluor mouse 594 and Alexa Fluor rabbit 488 diluted 1:1000. Immunofluorescence analysis was performed by ViCo (NIKON) or DM4000B (LEICA). For confocal image acquisition, an inverted Zeiss LSM880 with fast airyscan microscope was used. The setup was controlled by ZEN black (software version, Carl Zeiss Microscopy GmbH). The nucleolar fluorescence of ErbB3 after NRG1 stimulation was quantified following the instructions reported hereafter https://theolb.readthedocs.io/en/latest/imaging/measuring-cell-fluorescence-using-imagej.html

### Immunoblotting and Immunoprecipitation

Immunoblotting analysis were performed using the mouse monoclonal antibody anti-ErbB3 (RTJ2 THERMO-FISHER), mouse anti-actin (Santa Cruz), anti C23 (sc-8031 Santa Cruz Dallas, TX, USA), anti-tubulin (sc-5274 SANTA CRUZ BIOTECHNOLOGY Dallas, TX, USA). The secondary antibodies used are the anti-mouse and anti-rabbit (GE HEALTHCARE BIO-SCIENCES, Piscataway, NJ, USA). *Whole extract*. Whole extract were prepared from sub-confluent cultures by resuspending cells in RIPA-Buffer (20 mM Hepes, pH 6.8, 5 mM KCl, 5 mM MgCl_2_, 0.5% NP-40, 0.1% sodium deoxycholate, protease inhibitor (SIGMA), 0.1 mM phenylmethylsulfonyl fluoride) after incubation for 30 min at 4 °C the samples were centrifuged at 10000 rpm × 15 min 4 °C. *Nuclear extracts*: cells at 80% of confluence were washed twice with PBS, and incubated in NE1 buffer (10 mM Hepes pH 8.0, 1.5 mM MgCl_2_, 10 mM KCl, 1 mM DTT, 0,1 mM PMSF (phenylmethylsulfonyl fluoride) and 1 mM Na-orthovanadate for 15 min at 4 °C. Homogenization of the cells was performed using a Dounce homogenizer and the lysate was centrifuged at 3000 rpm × 5 min at 4 °C. The nuclear pellet was resuspended in NE2 buffer (20 mM Hepes pH 8.0, 1.5 mM MgCl_2_, 25% glycerol, 420 mM NaCl, 0.2 mM EDTA, 1 mM DTT, 1 mM Na-orthovanadate, 0.1 mM PMSF and incubated for 30 min at 4 °C. Finally, the supernatant was cleared by centrifuging for 2 min at 12.000 rpm. *Nucleolar extracts*: the nucleolar extracts were prepared as reported in Ponti et al. [4] and Avitabile et al. [45]**.** Specific antibodies for each compartments were used: anti Pol I subunit RPA40 (sc-101,126, SANTA CRUZ BIOTECHNOLOGY, Dallas, Tx, USA) for the nucleoli; anti GAPDH (ab181602, Abcam, Cambridge, MA, USA) for the cytoplasm and anti lamin B1 (33–2000, Thermo Fisher Scientific Waltham, Massachusetts, USA) for the nuclei. For experiments of immunoprecipitation, an equal amount of precleared HeLa and U-87MG nuclear extracts (150 μg) was immunoprecipitated with anti-ErbB3 antibody (RTJ2) or scramble antibodies (IgG cod n. 5409 Thermo Fisher) for 6 h at 4 °C in binding buffer (50 mM Tris pH 7.8 and 150 mM NaCl). The complex was pull down by using magnetic beads (Protein G, MERK, MILLIPORE, Darmstadt, Germany) according to Ponti et al. [4]. After incubation, the beads were washed three times with binding buffer and then with 1x PBS before the incubation at 95 °C for 10 min with 50 μl of 2XSDS loading buffer. The immunocomplex eluted was analysed by western blot as previously described by Ponti et al. [4, 5]. Protein extracts were resolved by 8% or 10% SDS-PAGE, blotted by semidry transfer apparatus (BIO-RAD) on 0.45 μM PVDF (AMERSHAM, Buckinghamshire, UK). The incubation with primary antibodies was performed overnight in 5% dry milk (CELL SIGNALING) at 4 °C (dilution 1:1000) followed by the incubation with secondary antibodies (AMERSHAM, Buckinghamshire, UK) (dilution 1:10000). The protein detection was performed by ECL reaction (AMERSHAM Buckinghamshire, UK) according to the manufacturer’s instructions. The images were acquired by ChemiDoc XRS+ with Image Lab Software (BIORAD) or revealed by Kodak film (AMERSHAM Hyperfilm ECL). All experiments have been done in triplicate. The Band quantification was performed with the ImageJ software and the data shown in the histograms represent the average of at least three independent experiments.

### qRT-PCR

Total RNA was extracted from cell lines by using Qiazol reagent (BIORAD). For cDNA preparation 1 μg of RNA was used for reverse transcription by SensiFast cDNA (BIOLINE) kit according to the manufacturer’s recommendations. The tissue of patients stored at − 80 °C in OCT (Optimal cutting temperature compound) and used for total RNA extraction by using the Qiagen FFPE plus mini kit (QIAGEN). The samples were processed by electrical homogenizer (IKA T-10 basic). cDNA preparation was performed by using the High capacity cDNA reverse transcription kit (APPLIED BIOSYSTEM). The quantification of total RNA was performed, by using Nanodrop one (Thermo Fischer). For silencing the ErbB3 pre-designed siRNA (INVITROGEN, Grand Island, NY USA cod. AM16708) or scrambled sequence RNA oligonucleotide, (INVITROGENE, Grand Island, NY USA cod. siRNA 4,390,846) were transiently transfected at 30 nM using Lipofectamine 2000 agent (INVITROGENE) according to the manufacturer’s instructions. Quantitative RT-PCR was performed using Fast SYBR Green Master mix and the StepOnePlus real-time PCR system (APPLIED BIOSYSTEMS). Each experiment was performed in triplicate and is expressed as mean 6 SEM. Experiments were independently repeated three times. Gene expression levels were quantified from real-time PCR data by the comparative threshold cycle (CT) method using 18S as an internal control gene according to Ponti et al. [4, 5]. All the experiments have been repeated at least three times.

47S: FW 5′-TGTCAGGCGTTCTCGTCTC-3′ and REV 5′-GAGAGCACGACGTCACCAC-3′;

18S: FW 5′-GCAATTATTCCCCATGAACG-3′ and REV 5′-GGGACTTAATCAACGCAAGC-3′; ErbB3: FW 5′-CAGGTTCAGGTGGCAGATTT-3′ and REV 5′-TCTCAAGGGCCATCCACTTA-3′; GFAP: FW 5′-AGCCACATCGCTCAGA CAC-3′ REV 5′-GCCCAATACGACCAAATCC-3′.

#### Cell cycle

For cell cycle analysis,1 × 10^6^ cells were fixed in 70% ethanol for 24 h, incubated with 50 μg/ml propidium iodide (PI, SIGMA-ALDRICH) and 50 units/ml DNase-free RNase A (SIGMA-ALDRICHigma-Aldrich) and analysed after 3 h using an Epics XL Cytometer (BECKMAN COULTER, Brea, CA). Each experiment was performed independently three times.

### Immunohistochemistry

The immunohistochemistry was conducted as previously described by Rosa et al. [46] with some modifications. In Brief, paraffin-embedded tissues of 2 GBM patients were deparaffinized in xylene, rehydrated in descending graded alcohols, incubated for 15 min in 3% H2O2 in methanol to block the activity of endogenous peroxidase activity, and then subjected to heat-induced antigen retrieval for 20 min in sodium citrate buffer (10 mM tri-sodium citrate dihydrate, 0.05% Tween 20, pH 6.0). After a blocking step with normal horse serum for 1 h, sections were incubated overnight with mouse anti-ErbB3 (sc-17, dilution 1:200) at 4 °C, washed three times with PBS, incubated for 1 h at room temperature with biotinylated universal secondary antibody (Vectastain Universal Elite ABC kit, Vector Laboratories, Burlingame, CA), washed three times with PBS and then incubated with the avidin–biotin-peroxidase complex according to the manufacturer’s instructions. Slides were successively stained with 3–3-diaminobenzidine (ImmPACT DAB peroxidase substrate, Vector Laboratories, Burlingame, CA) to visualize the reaction product, and were counterstained with hematoxylin to visualize nuclei. A Nikon Eclipse Ni motorized microscope system at 20x magnification was used to acquire images. This study was carried out according to the principles of the Helsinki Declaration and the protocols approved by the ethics committee.

### Statistical analysis

Each experiment was repeated at least three times. Statistical procedures were performed using Graphpad Prism 5 software. Statistical comparisons were performed using Student’s *t*-test and a one-way analysis of variance (ANOVA). A *P*-value of 0.05 or lower considered to be statistically significant.

## Supplementary Information

**Additional file 1: Supplementary Figure 1.** (A) ErbB3 localizes in the nucleolus of HeLa and MCF-7 cells. Immunofluorescence analysis for ErbB3 (green) and Fibrillarin (red) after 24h of serum starvation (0.2% FBS). (B). Confocal magnifications and relative lateral sections are shown HeLa cells. (C) ErbB3 localization in MCF-7. The staining was performed using rabbit polyclonal anti-ErbB3 C17 (green), anti Pol I subunit RPA40 (red). The colocalization is shown in yellow. Scalebar represent 10 μm in A and 25 μm in C.

**Additional file 2: Supplementary Figure 2.** (A) Immunofluorescence of U-87MG without anti-ErbB3 primary antibody. Cells were treated only with the secondary antibody Alexa Flour 488 (secondary antibody in green and dapi in blue). No nucleolar specific signal was detected. (B) Immunofluorescence of U-87MG without permeabilization. Cells were treated with primary (anti-ErbB3) and secondary (Alexa Fluor 488) without the permeabilization step. (ErbB3 in green and dapi in blue). No nucleolar specific signal was detected.

**Additional file 3: Supplementary Figure S3.** ErbB3 localizes in the nucleolus of U-87MG probed with an anti ErbB3 RTJ2 monoclonal antibody after 24h of serum starvation (0.2% FBS). ErbB3 is shown in green and Fibrillarin in red. Scalebar represent 5 μm.

**Additional file 4: Supplementary Figure 4.** The 50 kDa variant of ErbB3 is detected in MCF-7 cells. (A) SDS-page of whole cells lysate from U-87MG and MCF-7. Approximatively 40 μg were loaded on each lane. (B) Quantification of ErbB3 expression. Data is the mean of three independent experiments. (** *p* <0.01).

**Additional file 5: Supplementary Figure S5.** HeLa cells were subjected to cell fractionation after 15 min of Neuregulin1 stimulation and analysed by western blot.

**Additional file 6: Supplementary Figure 6.** Difference in medium pH of U-87MG after 72h exposure to actinomycin D.

**Additional file 7: Supplementary Figure 7.** Immunohistochemistry of glioblastoma patient biopsies. ErbB3 is stained in brown and the nuclei are in blue.

**Additional file 8:.** Supplementary information.

## Data Availability

All data generated or analysed during this study are included in this published article. .

## Abbreviations

ErbB3: Receptor tyrosine-protein kinase erb3
UBF: Upstream binding factor
siErbB3: Silencing ErbB3
EGFR: Epidermal growth factor receptor
ARF: Alternate reading frame protein
EGR1: Early growth response 1
ActD: Actinomycin D
GFAP: Glial fibrillary acidic protein
ErbB3: v-erb-b erythroblastic leukemia viral oncogene homolog 3
