# Supplementary material for: Nucleolar localization of the ErbB3 receptor as a new target in glioblastoma
Source: BMC Mol Cell Biol. 2022 Mar 7;23:13. doi: 10.1186/s12860-022-00411-y (PMC8900349; doi:10.1186/s12860-022-00411-y)
Supplement: Supplementary file 1 — Additional file 1: Supplementary Figure 1. (A) ErbB3 localizes in the nucleolus of HeLa and MCF-7 cells. Immunofluorescence analysis for ErbB3 (green) and Fibrillarin (red) after 24h of serum starvation (0.2% FBS). (B). Confocal magnifications and relative lateral sections are shown HeLa cells. (C) ErbB3 localization in MCF-7. The staining was performed using rabbit polyclonal anti-ErbB3 C17 (green), anti Pol I subunit RPA40 (red). The colocalization is shown in yellow. Scalebar represent 10 μm in A and 25 μm in C. [file 12860_2022_411_MOESM1_ESM.pdf]

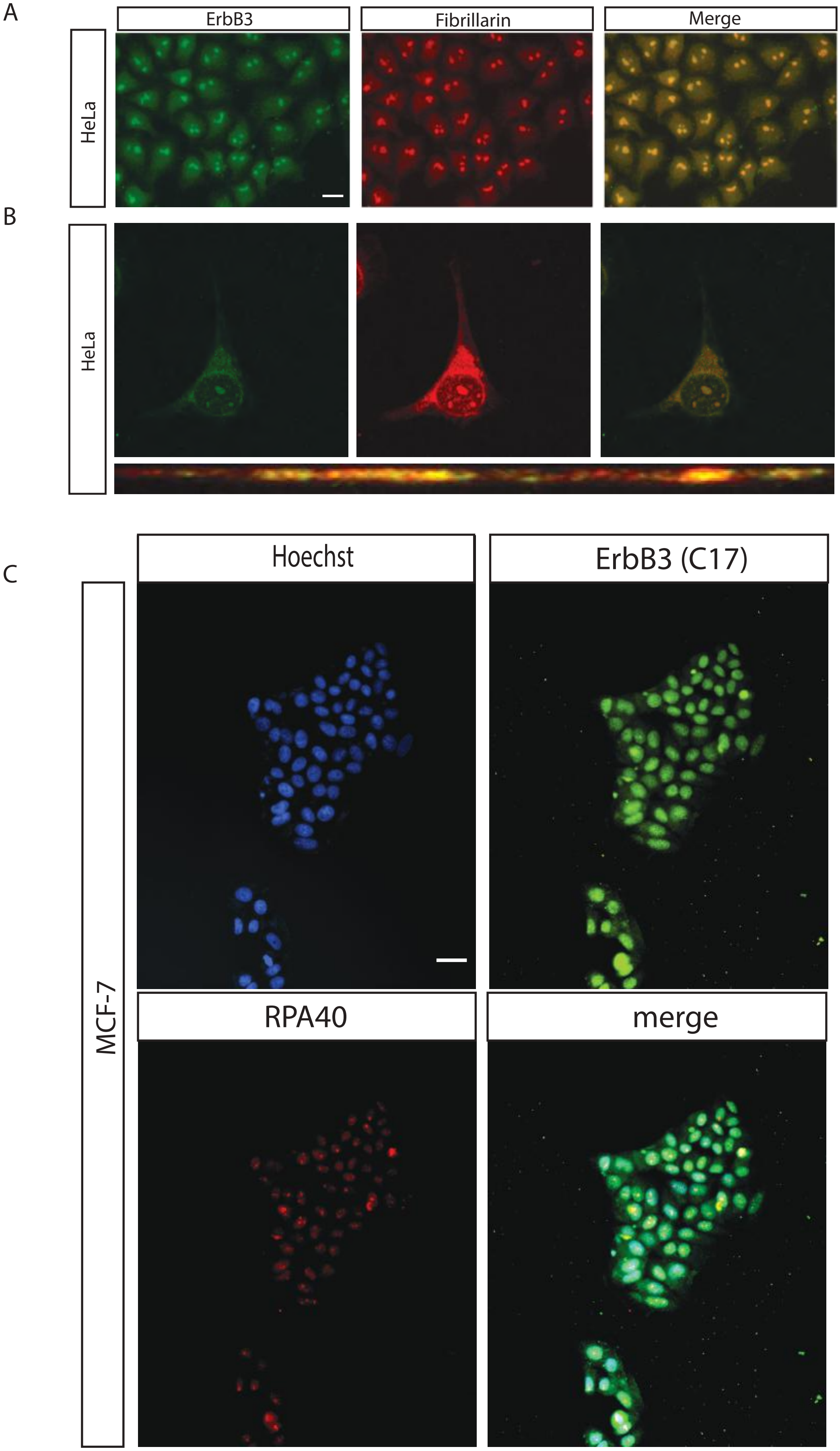

Supplementary Figure 1. (A) ErbB3 localizes in the nucleolus of HeLa and MCF-7 cells. Immunofluorescence analysis for ErbB3 (green) and Fibrillarin (red) after 24h of serum starvation (0.2% FBS). (B). Confocal magnifications and relative lateral sections are shown HeLa cells. (C) ErbB3 localization in MCF-7. The staining was performed using rabbit polyclonal anti-ErbB3 C17 (green), anti Pol I subunit RPA40 (red). The colocalization is shown in yellow. Scalebar represent 10 μm in A and 25 μm in C.
