# Supplementary material for: Nucleolar localization of the ErbB3 receptor as a new target in glioblastoma
Source: BMC Mol Cell Biol. 2022 Mar 7;23:13. doi: 10.1186/s12860-022-00411-y (PMC8900349; doi:10.1186/s12860-022-00411-y)
Supplement: Supplementary file 2 — Additional file 2: Supplementary Figure 2. (A) Immunofluorescence of U-87MG without anti-ErbB3 primary antibody. Cells were treated only with the secondary antibody Alexa Flour 488 (secondary antibody in green and dapi in blue). No nucleolar specific signal was detected. (B) Immunofluorescence of U-87MG without permeabilization. Cells were treated with primary (anti-ErbB3) and secondary (Alexa Fluor 488) without the permeabilization step. (ErbB3 in green and dapi in blue). No nucleolar specific signal was detected. [file 12860_2022_411_MOESM2_ESM.pdf]

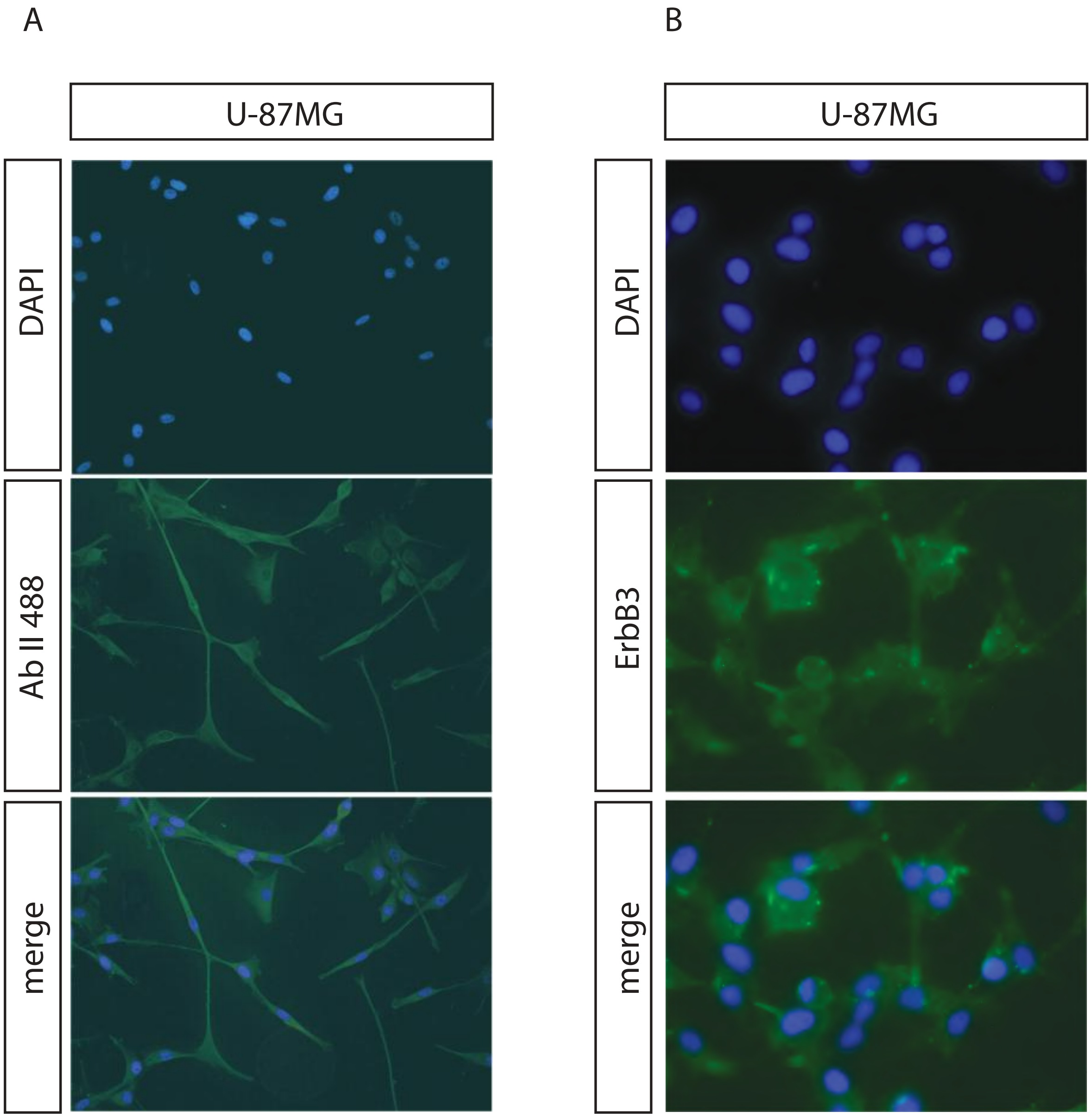

Supplementary Figure 2. (A) Immunofluorescence of U-87MG without anti-ErbB3 primary antibody. Cells were treated only with the secondary antibody Alexa Flour 488 (secondary antibody in green and dapi in blue). No nucleolar specific signal was detected. (B) mmunofluorescence of U-87MG without permeabilization. Cells were treated with primary (anti-ErbB3) and secondary (Alexa Fluor 488) without the permeabilization step. (ErbB3 in green and dapi in blue). No nucleolar specific signal was detected.
