# Supplementary material for: Nucleolar localization of the ErbB3 receptor as a new target in glioblastoma
Source: BMC Mol Cell Biol. 2022 Mar 7;23:13. doi: 10.1186/s12860-022-00411-y (PMC8900349; doi:10.1186/s12860-022-00411-y)
Supplement: Supplementary file 3 — Additional file 3: Supplementary Figure S3. ErbB3 localizes in the nucleolus of U-87MG probed with an anti ErbB3 RTJ2 monoclonal antibody after 24h of serum starvation (0.2% FBS). ErbB3 is shown in green and Fibrillarin in red. Scalebar represent 5 μm. [file 12860_2022_411_MOESM3_ESM.pdf]

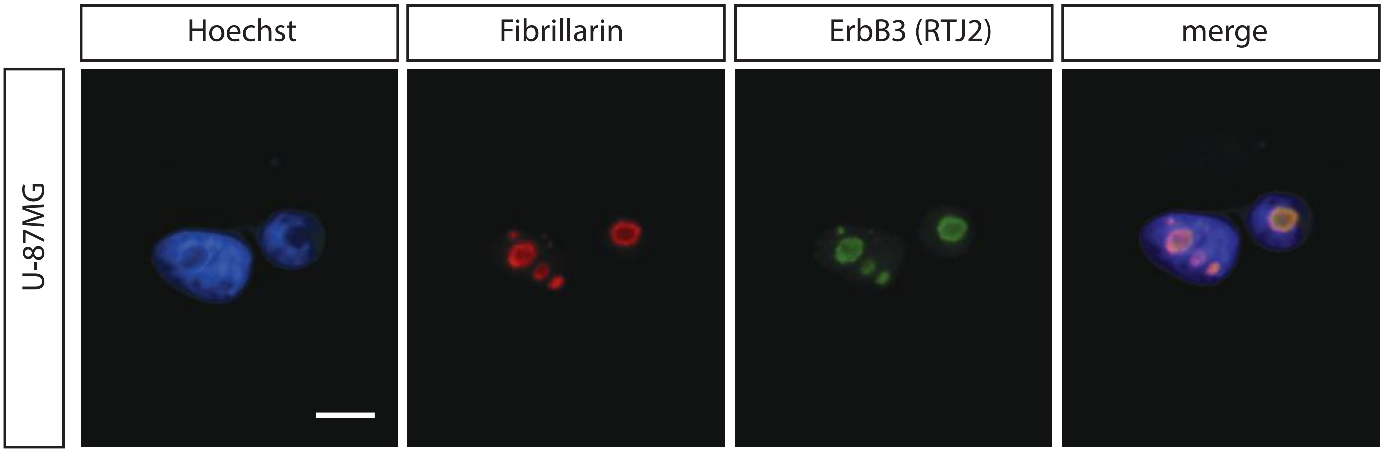

Supplementary figure S3. ErbB3 localizes in the nucleolus of U-87MG probed with an anti ErbB3 RTJ2 monoclonal antibody after 24h of serum starvation (0.2% FBS). ErbB3 is shown in green and Fibrillarin in red. Scalebar represent 5 µm.
