# Supplementary material for: Nucleolar localization of the ErbB3 receptor as a new target in glioblastoma
Source: BMC Mol Cell Biol. 2022 Mar 7;23:13. doi: 10.1186/s12860-022-00411-y (PMC8900349; doi:10.1186/s12860-022-00411-y)
Supplement: Supplementary file 4 — Additional file 4: Supplementary Figure 4. The 50 kDa variant of ErbB3 is detected in MCF-7 cells. (A) SDS-page of whole cells lysate from U-87MG and MCF-7. Approximatively 40 μg were loaded on each lane. (B) Quantification of ErbB3 expression. Data is the mean of three independent experiments. (** p <0.01). [file 12860_2022_411_MOESM4_ESM.pdf]

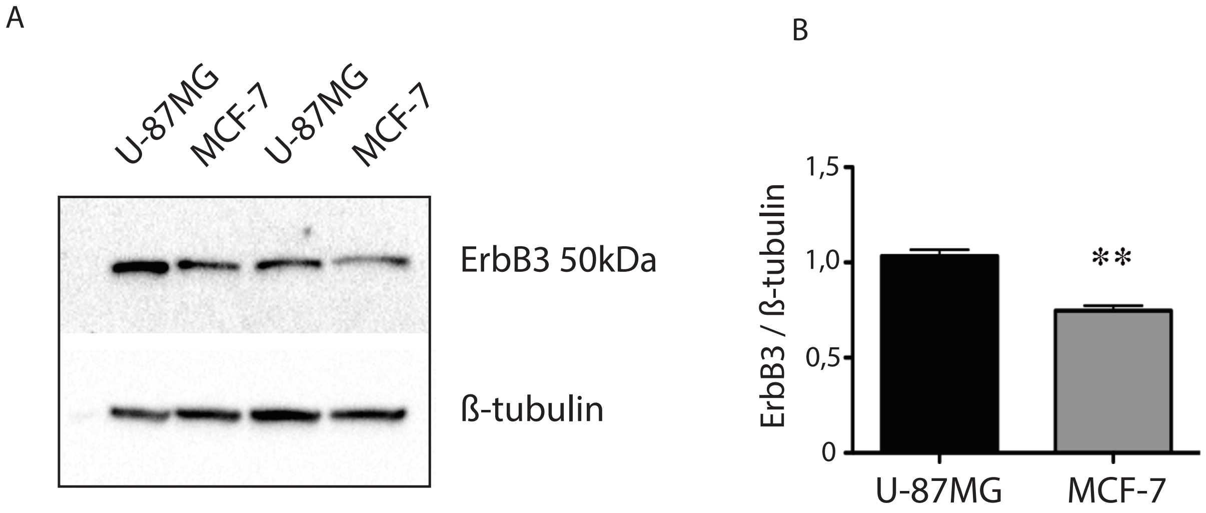

Supplementary figure 4: The 50 kDa variant of ErbB3 is detected in MCF-7 cells. (A) SDS-page of whole cells lysate from U-87MG and MCF-7. Approximatively 40 µg were loaded on each lane. (B) Quantification of ErbB3 expression. Data is the mean of three independent experiments. (** $p<0.01$).
