# Supplementary material for: Nucleolar localization of the ErbB3 receptor as a new target in glioblastoma
Source: BMC Mol Cell Biol. 2022 Mar 7;23:13. doi: 10.1186/s12860-022-00411-y (PMC8900349; doi:10.1186/s12860-022-00411-y)
Supplement: Supplementary file 5 — Additional file 5: Supplementary Figure S5. HeLa cells were subjected to cell fractionation after 15 min of Neuregulin1 stimulation and analysed by western blot. [file 12860_2022_411_MOESM5_ESM.pdf]

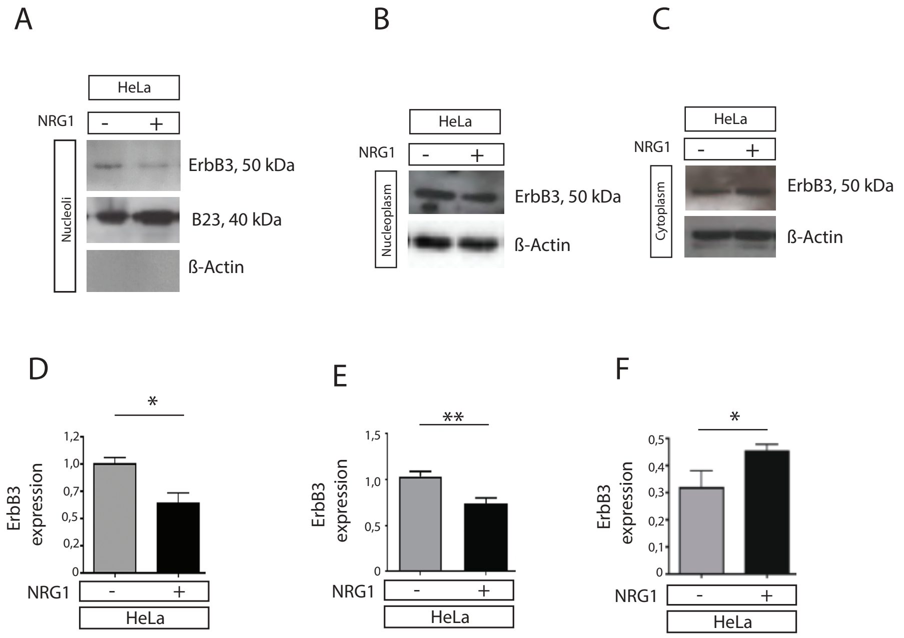

Supplementary figure S5. HeLa cells were subjected to cell fractionation after 15 min of Neuregulin1 stimulation and analysed by western blot. Purified nucleoli (A), Nucleoplasm (B) and cytoplasmic extracts (C) were probes with anti ErbB3, anti B23 (only for nucleoli) and anti ß-actin antibodies. The absence of actin is a control for nucleolar extraction efficiency. Densitometric quantification of the ErbB3 signal was performed by Image J in the nucleoli (D), nucleoplasm (E) and cytoplasmic extract (F). Data are the average of three independent experiments and significant results are highlighted with asterisks (* $p<0.05$, ** $p<0.01$).
