# Supplementary figures and images for: Nucleolar localization of the ErbB3 receptor as a new target in glioblastoma
Source: BMC Mol Cell Biol. 2022 Mar 7;23:13. doi: 10.1186/s12860-022-00411-y (PMC8900349; doi:10.1186/s12860-022-00411-y)

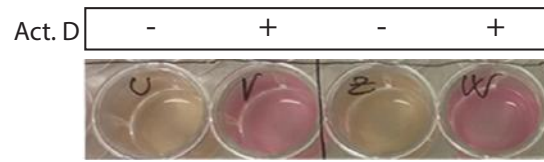

Supplementary figure 6. Difference in medium pH of U-87MG after 72h exposure to actinomycin D.

Supplement: Supplementary file 6 — Additional file 6: Supplementary Figure 6. Difference in medium pH of U-87MG after 72h exposure to actinomycin D. [file 12860_2022_411_MOESM6_ESM.pdf]
