# Supplementary material for: Nucleolar localization of the ErbB3 receptor as a new target in glioblastoma
Source: BMC Mol Cell Biol. 2022 Mar 7;23:13. doi: 10.1186/s12860-022-00411-y (PMC8900349; doi:10.1186/s12860-022-00411-y)
Supplement: Supplementary file 7 — Additional file 7: Supplementary Figure 7. Immunohistochemistry of glioblastoma patient biopsies. ErbB3 is stained in brown and the nuclei are in blue. [file 12860_2022_411_MOESM7_ESM.pdf]

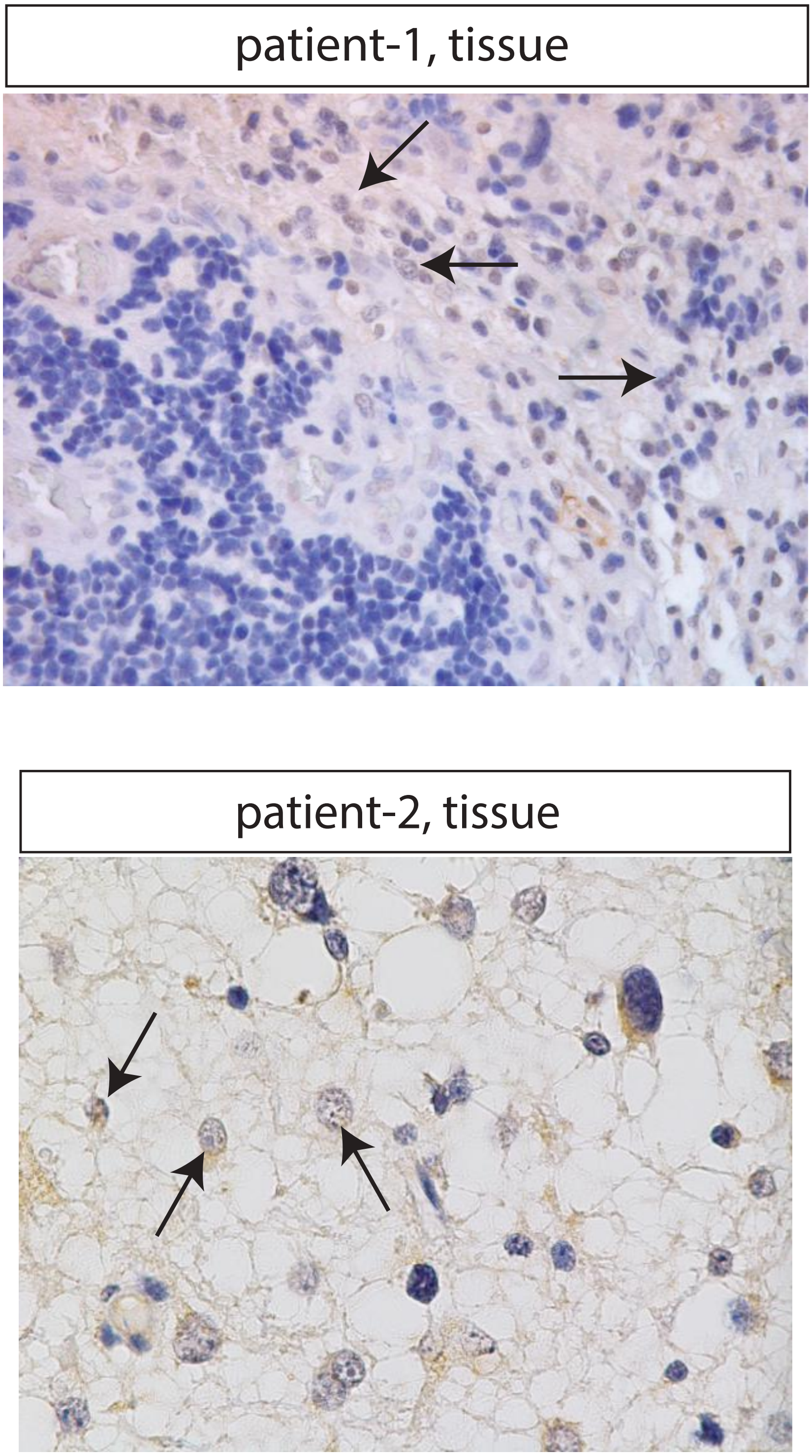

Supplementary Figure 7. Immunohistochemistry of glioblastoma patient biopsies.
ErbB3 is stained in brown and the nuclei are in blue.
