# Supplementary material for: Nucleolar localization of the ErbB3 receptor as a new target in glioblastoma
Source: BMC Mol Cell Biol. 2022 Mar 7;23:13. doi: 10.1186/s12860-022-00411-y (PMC8900349; doi:10.1186/s12860-022-00411-y)
Supplement: Supplementary file 8 — Additional file 8:. Supplementary information. [file 12860_2022_411_MOESM8_ESM.pdf]

Supplementary Information

Nucleolar localization of the ErbB3 receptor as a new target in glioblastoma

Marzia Tagliaferro[1°], Paolo Rosa[1°], Gian Carlo Bellenchi[2,3,4] Daniela Bastianelli[5], Rosa Trotta[6,7], Claudia Tito[8], Francesco Fazi[8], Antonella Calogero[1,5], Donatella Ponti[1,6*].

[1]Department of Medical-Surgical Sciences and Biotechnologies, University of Rome Sapienza, 04100, Latina, Italy.
[2]Institute of Genetics and Biophysics “Adriano Buzzati Traverso” CNR Naples 80131 Italy.
[3]Fondazione Santa Lucia IRCCS, 00143 Rome, Italy.
[4]University of Tor Vergata, Department of Systems Medicine, 00133 Rome, Italy.
[5]Istituto Chirurgico Ortopedico Traumatologico, Latina 04100, Italy.
[6]Laboratory of Tumor Inflammation and Angiogenesis, Center for Cancer Biology (CCB), VIB, Leuven, Belgium.
[7]Laboratory of Tumor Inflammation and Angiogenesis, and Department of Oncology, KU Leuven, Belgium
[8]Department of Anatomical, Histological, Forensic and Orthopaedic Sciences, Sapienza University of Rome, 00185, Rome, Italy.

[°]These authors contributed equally to this work.

Correspondence to:
*Donatella Ponti, PhD
University of Rome La Sapienza
Corso della Repubblica 79, 04100 Latina, Italy.
E-Mail: donatella.ponti@uniroma1.it

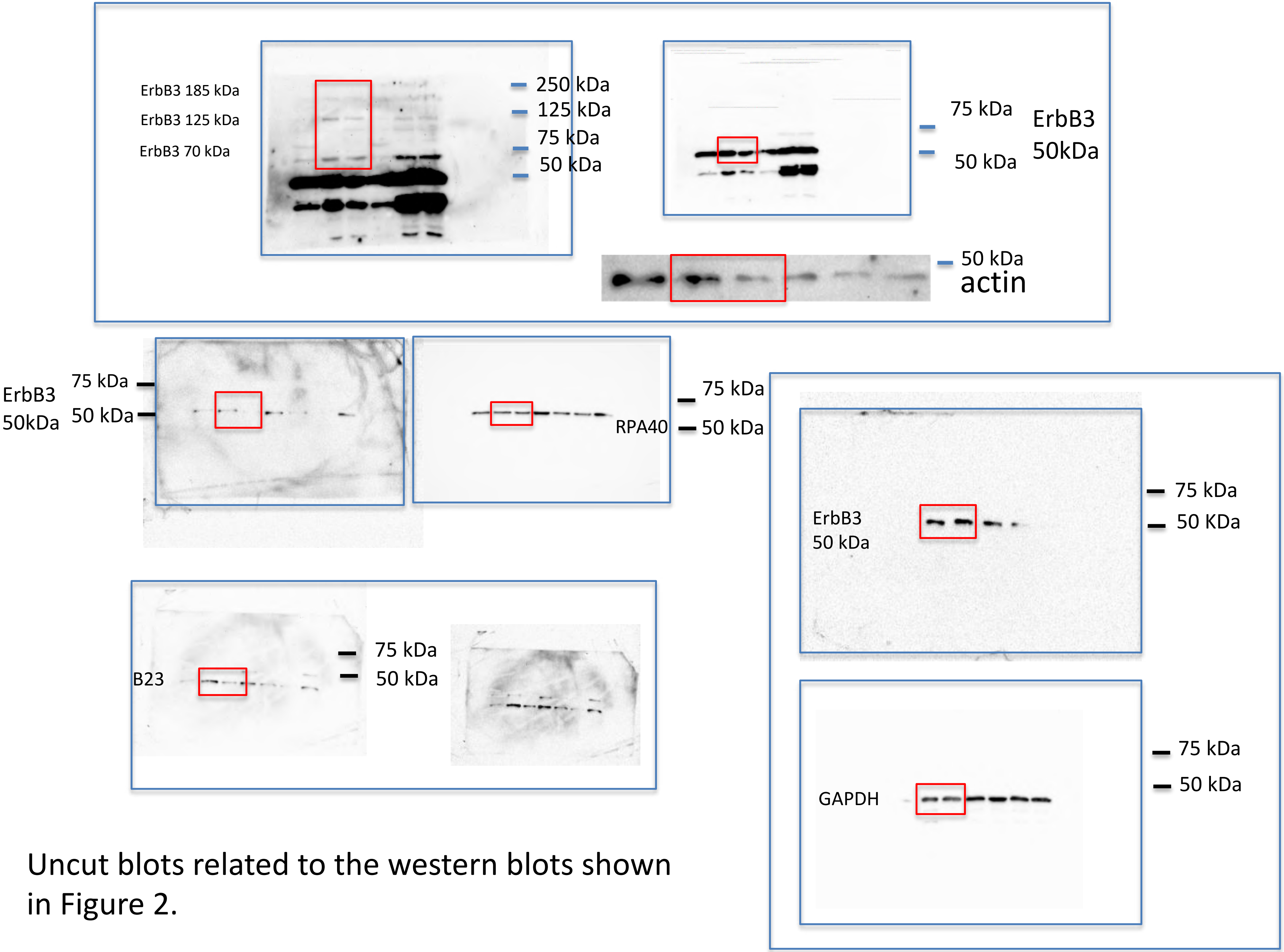

Uncut blots related to the western blots shown in Figure 2.

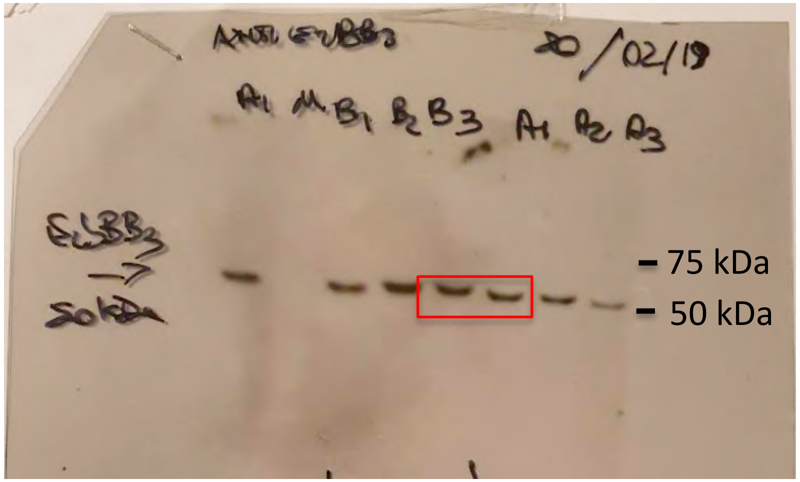

ErbB3 50 kDa

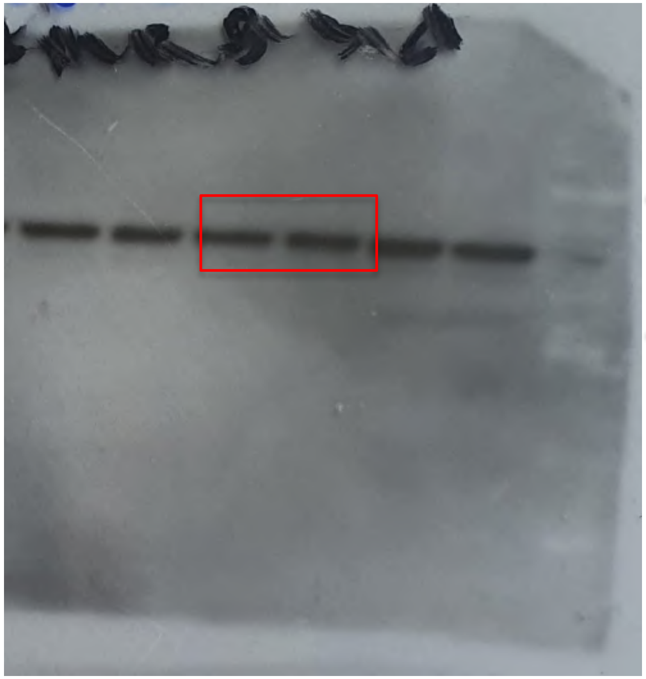

75 kDa

Lamin B

50 kDa

Uncut blots related to the western blots shown in Figure 2.

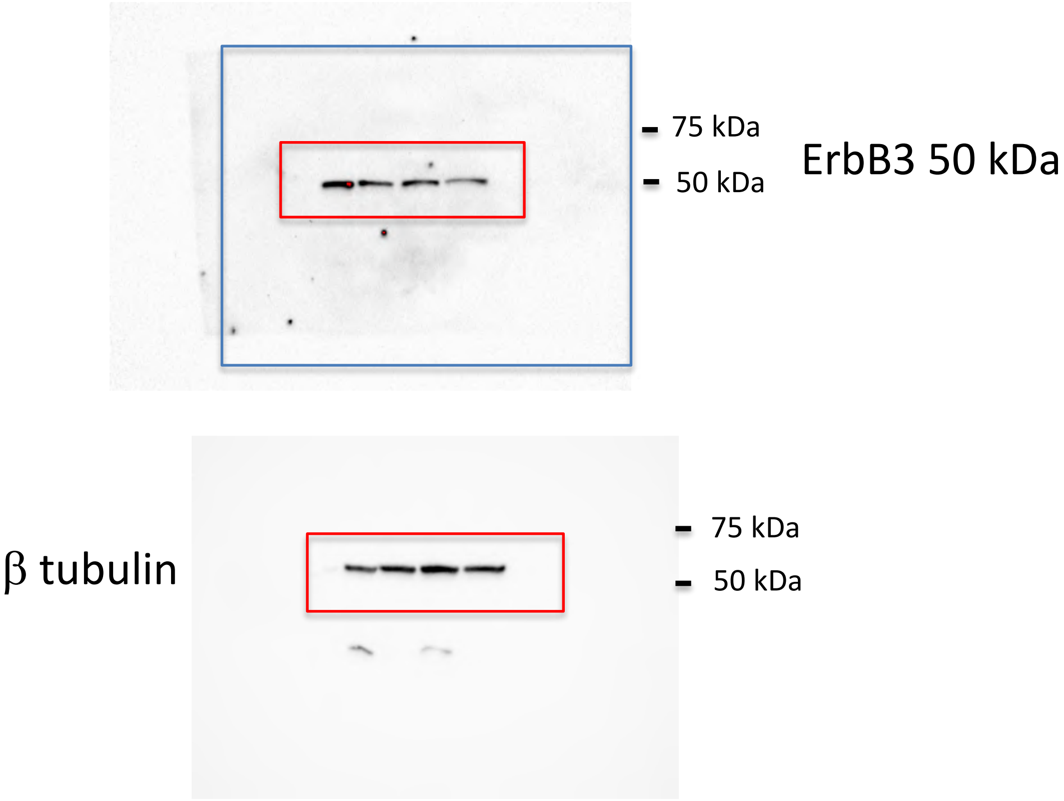

Uncut blots relative to the western blot on whole extract of U-87MG and MCF-7 cells shown in supplementary figure S4 .

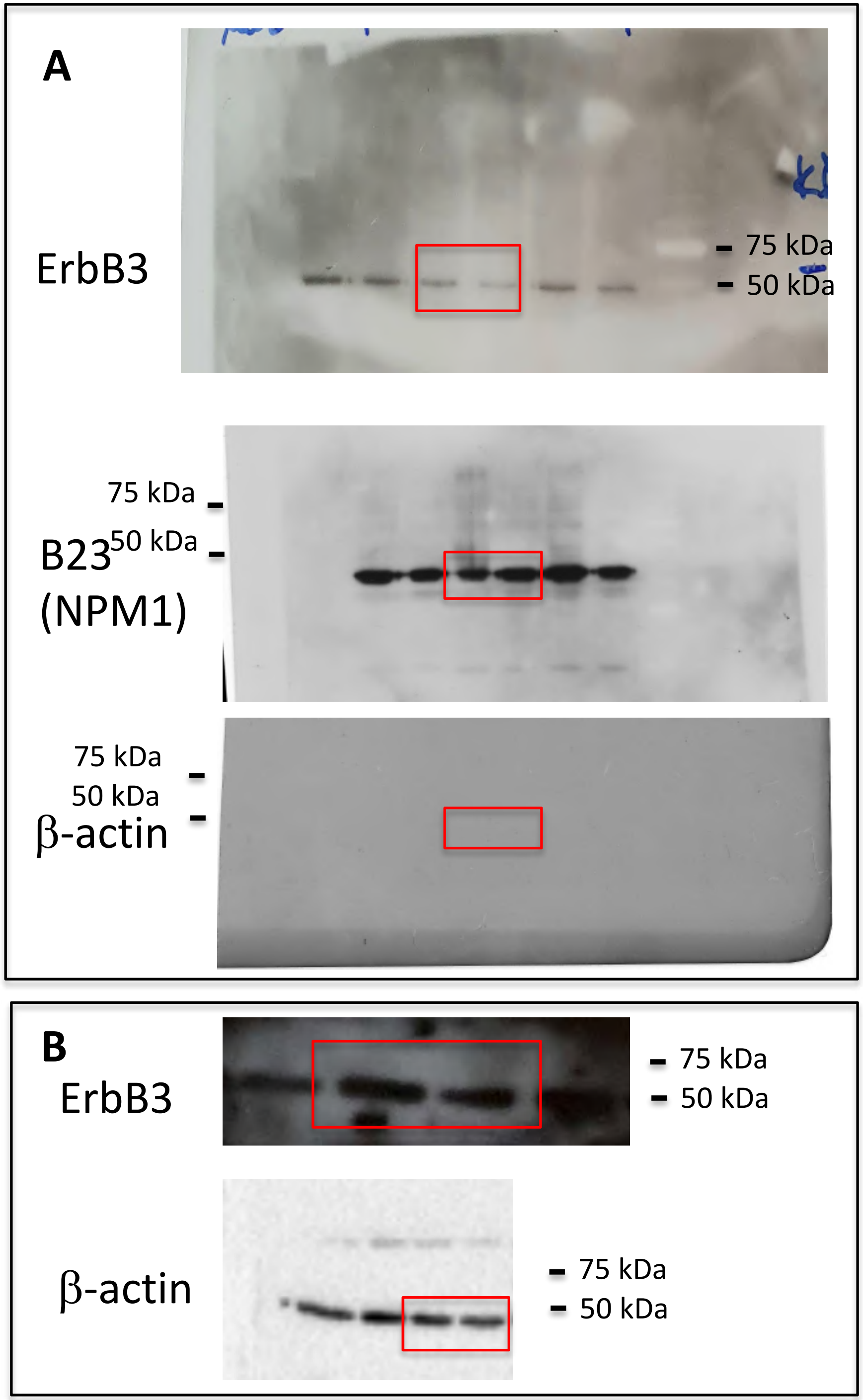

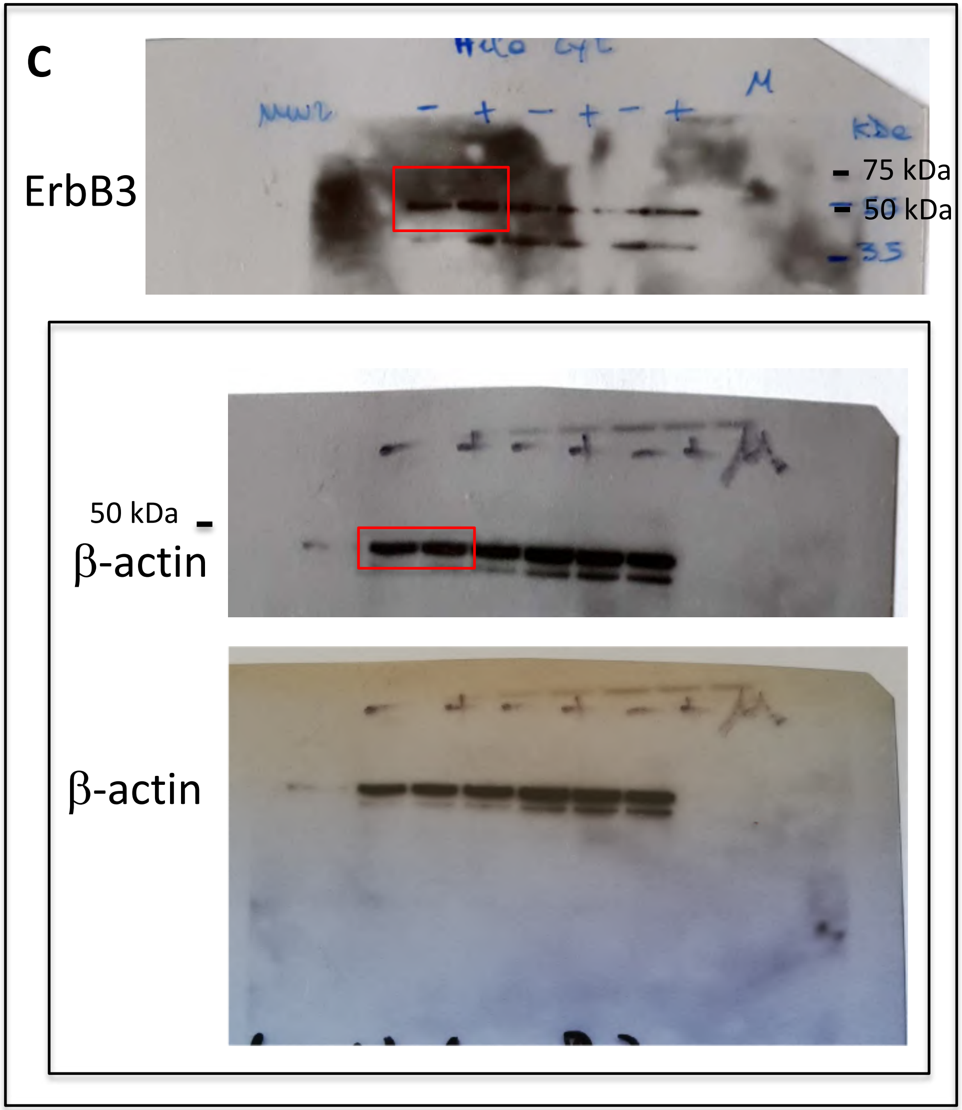

. Uncut blots relative to the western blots shown in Supplementary figure S5.

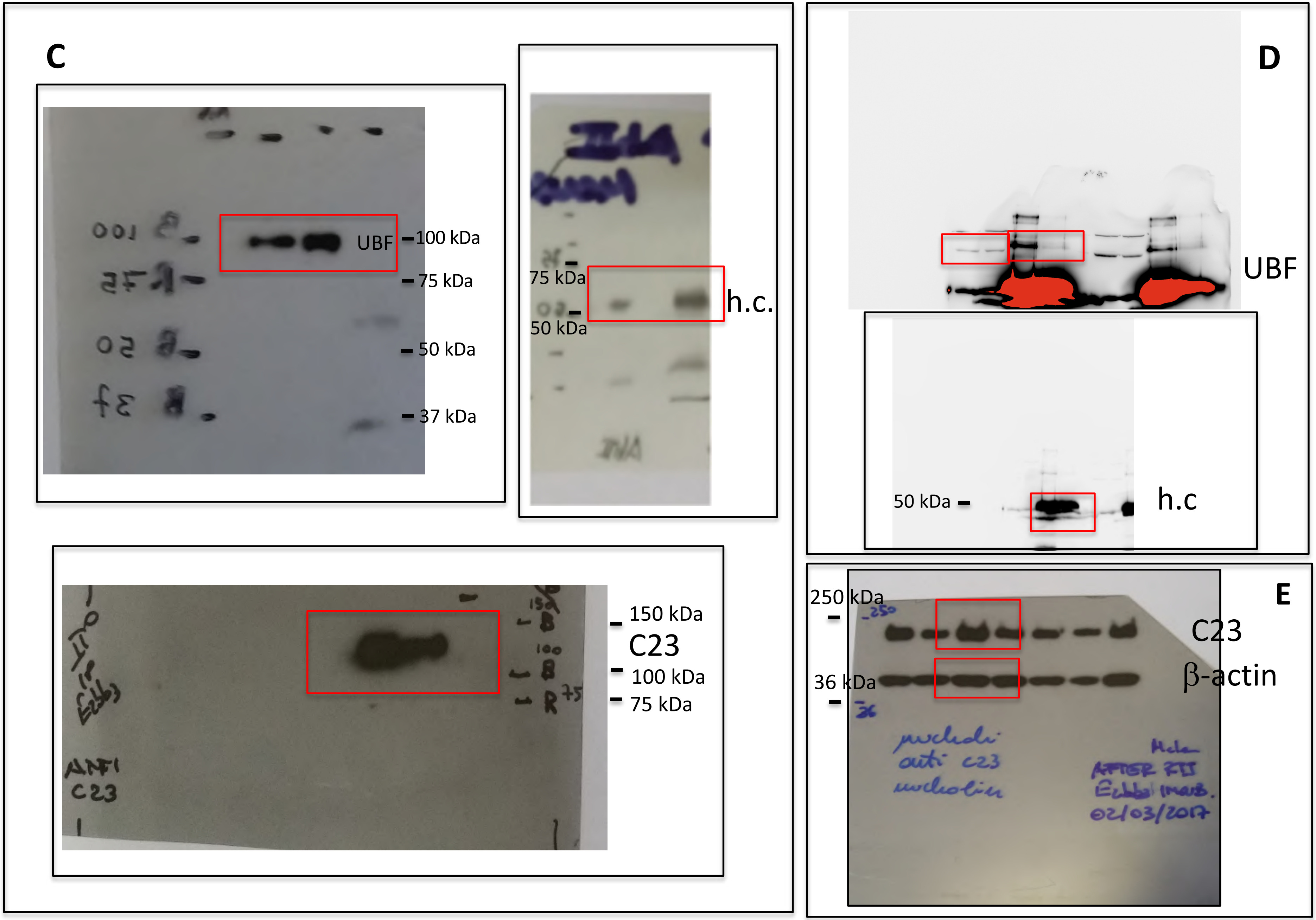

Uncut blots relative to the western blots shown in Figure 3

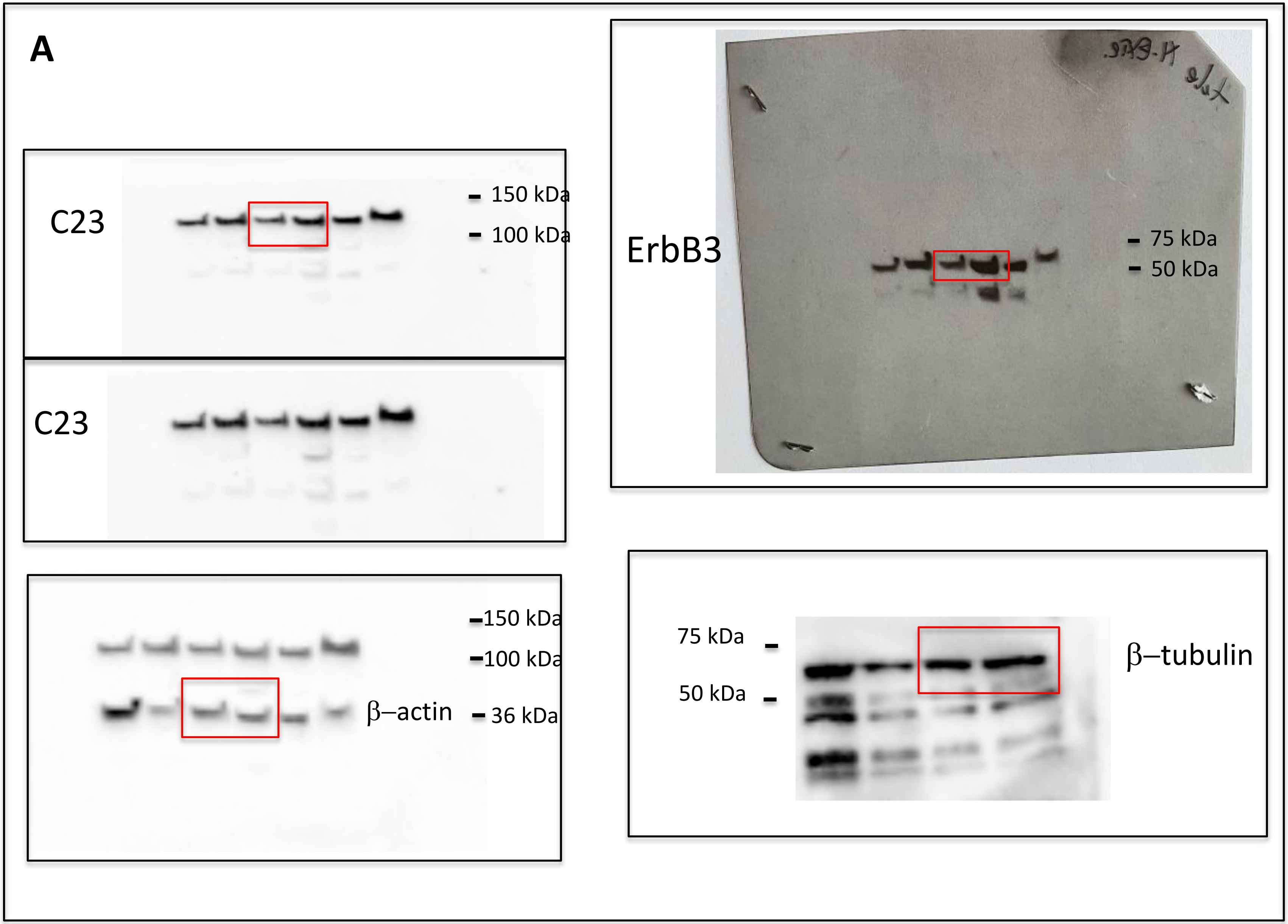

Uncut blots relative to the left panel of the western blots shown in Figure 4.

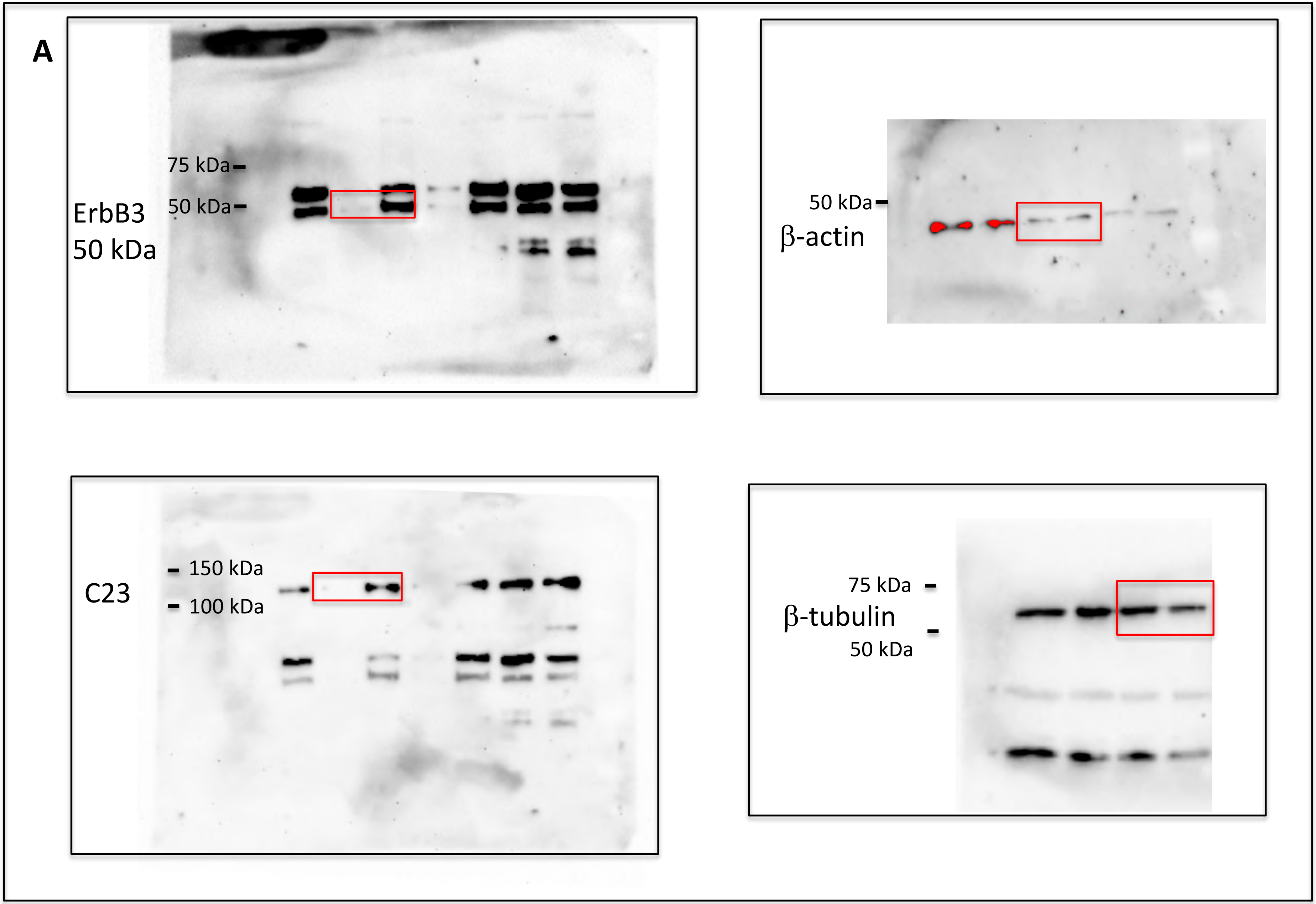

Uncut blots relative to the right panel of the western blots shown in Figure 4.

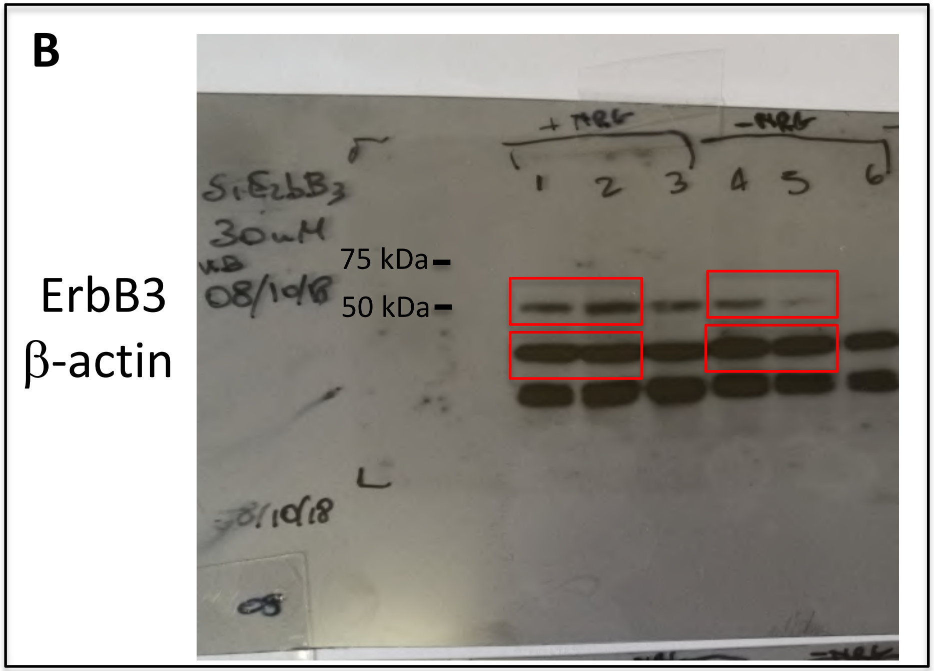

Uncut blots relative to the western blots shown in Figure 5.

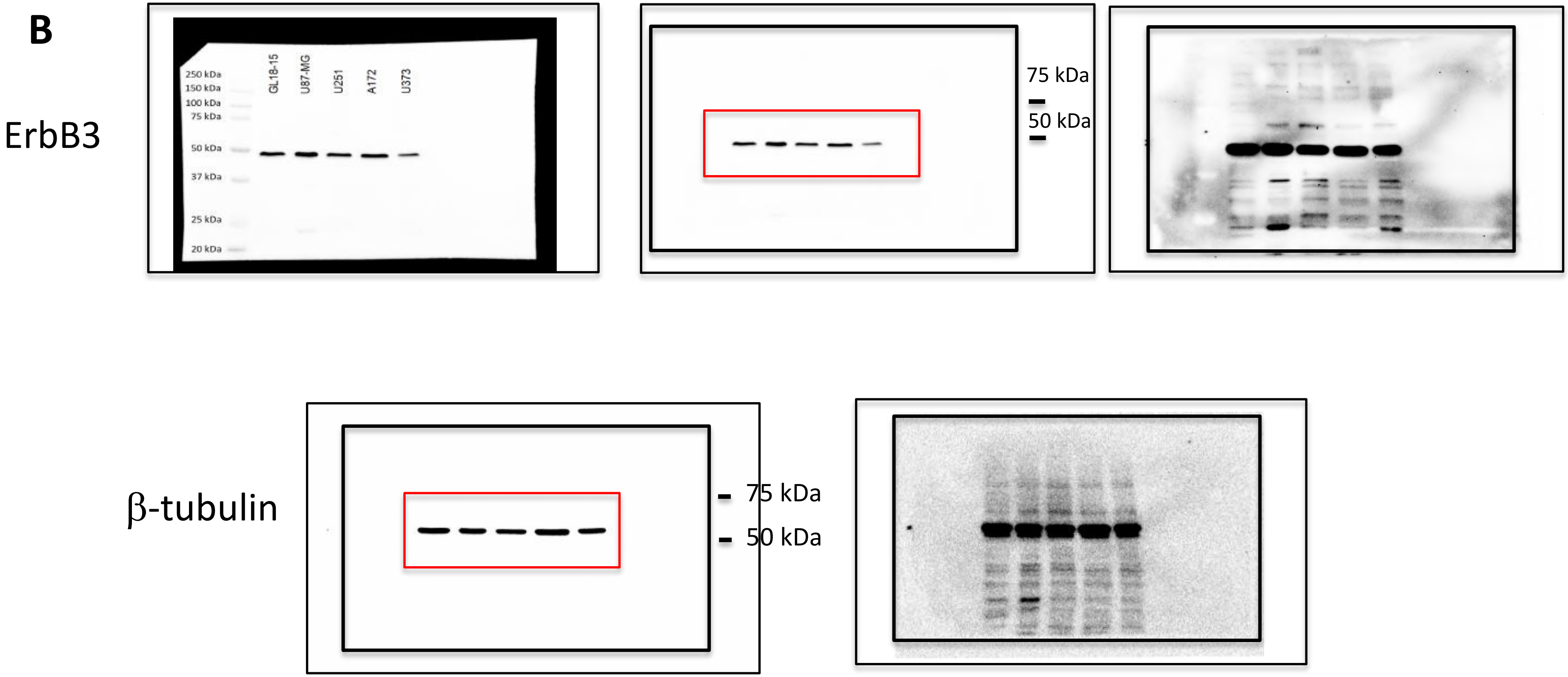

Uncut blots relative to the western blots shown in Figure 6.
